# Effect of microwave irradiation cycles on the pore structures and fractal dimension of coals with different ranks

**DOI:** 10.1371/journal.pone.0343007

**Published:** 2026-07-29

**Authors:** Liankun Zhang, Zizheng Wang, Xiaoyu Zhang, Geng Li, Bo Yin, Xiaomin Liang, Rongrong Duan, Yuefang Wang

**Affiliations:** 1 College of Resources and Environmental Engineering, Inner Mongolia University of Technology, Hohhot, China; 2 Emergency Science Research Academy, Chinese Institute of Coal Science, Beijing, China; 3 Analysis Test and Experiment Management Center, Inner Mongolia University of Technology, Hohhot, China; Northeastern University, CHINA

## Abstract

A better understanding of the changes in the coal pore structure and the fractal dimension after microwave irradiation is beneficial for studying the reservoir stimulation and thus enhanced coal bed methane (ECBM) extraction. Place coal samples of three different ranks, namely subbituminous coal, bituminous coal, and anthracite coal, under microwave irradiation (2.45 GHz) for 1, 3, 5, and 10 cycles, respectively. Low temperature nitrogen gas adsorption (LT-N_2_GA) and mercury intrusion porosimetry (MIP) were employed to characterize pore structure changes before and after irradiation cycles quantitatively, and fractal theory was applied to analyze the overall roughness and irregularity index of pores. The results show that the overall type of pores in three kinds of coal samples does not change after microwave irradiation cycles modification, but only changes the pore volume distribution of the coal samples. The total pore volume of coal samples was increased by microwave irradiation cycles; The pore volume and specific surface area of micropores (<10 nm) decreased; The increase of pore volume and specific surface area of small pores (10–50 nm); The surface irregularities and irregularities in pore size distribution of micropores and small pores are reduced, and this phenomenon further weakens with the increase in the number of cycles. With more cycles of microwave irradiation, the pore volume of macropores (>50 nm) in coal increases, while their specific surface area decreases. After irradiation, the size distribution irregularity of macropores (50 nm-20 μm) is reduced, whereas that of macropores (>20 μm) is increased. Microwave irradiation cycles promote the development, expansion, and connectivity of coal sample pores and fractures, and make some micropores expand into small pores, which increases and enhances the connectivity of macroporous fractures.

## Introduction

In the process of global energy development, the efficient and clean utilization of coal has always been a hot topic in the international community [1]. China’s coal is generally characterized by deep burial depth, large thickness, and complex reservoir conditions, yet the total coal bed methane (CBM) resources are abundant [2,3]. In recent years, China has made new breakthroughs in coal bed methane selection evaluation technology, drilling techniques, fracturing techniques, and drainage and production methods. Coal bed methane extraction has become a major part of coal resource development [4]. However, the low porosity, poor permeability, and strong methane adsorption capacity of coal reservoirs have restricted the efficient development of CBM [5–8]. Although conventional hydraulic and chemical solvent reservoir modification methods [9,10] have achieved certain results, the low modification efficiency and the environmental pollution they cause have limited their wide application [11]. Microwave has the characteristics of instantaneity, strong penetration, selectivity, controllability, and the synergistic effect of thermal and non-thermal effects [12–14]. In addition, microwave irradiation can change the pore structure, molecular structure, and methane adsorption and desorption of coal, so it is possible to strengthen the extraction of coal bed methane by microwave irradiation cycles [15–17]. Dong et al. [18] studied the effect of microwave on coal gasification by using synchronous thermal analyzer, Fourier transform infrared spectrometer, and quantum chemical calculation. The results showed that microwave drying could increase the gasification reactivity of coal and improve the performance of underground coal gasification. Qi et al. [19] studied the damage and seepage characteristics of water-saturated coal under the action of microwave. The results showed that microwave treatment had a good effect on enhancing permeability, and the initial permeability, failure permeability, and average permeability of coal samples were improved. Huang et al. [20] conducted a comparative study on microwave and conventional pyrolysis of tar-rich coal. They found that microwave irradiation excites oxygen-containing groups and aromatic rings in coal, generating heat that cleaves macromolecules into smaller compounds and promotes radical formation. Additionally, microwave pyrolysis reduces the oxygen content in tar through functional group migration and accelerates aliphatic conversion to radicals for H_2_/CO. According to Chemerinskiy et al. [21], the use of microwave technology may modify the coal metamorphic stage, which in turn enhances coal processing efficiency, lowers energy consumption, and minimizes hazardous emissions. Most existing studies focus on the macro phenomena of microwave treatment on coal, such as the improvement of permeability, gasification efficiency, pyrolysis characteristics, and coal rank transformation. However, the pore structures evolution of coal under microwave irradiation, especially the effect of different microwave cycles on the pore structures of different coal ranks, has not been fully studied.

The pore structure of coal is a key factor affecting the storage, diffusion, and exploitation of coal bed methane, and fractal theory combined with the low temperature N_2_ adsorption method (LT-N_2_GA) and mercury injection method is a good method to characterize the pore structure. Zhu et al. [22] used low temperature N_2_ adsorption and the Frenkel-Halsey-Hill (FHH) model to measure and study the structure and fractal characteristics of micropores and mesopores in coal samples during steam activation. Wang et al. [23] systematically studied the evolution and fractal characteristics of the pore structure of sandstone uranium ore after leaching at different temperatures by using the mercury intrusion method (MIP), low temperature N_2_ adsorption method, and scanning electron microscope (SEM). The results showed that compared with other leaching conditions, the N_2_ adsorption and mercury intrusion volume of the ore were the largest, the pore volume was more prominent, and the pore structure was more developed at 40 °C. Zha et al. [24] analyzed the pore structure characteristics of lignite, bituminous coal, and anthracite by the mercury intrusion method, liquid N_2_ adsorption method, and infrared spectroscopy, and analyzed the dynamic adsorption process of water vapor in different pore structures of coal samples. Liu et al. [25] established a coal gas seepage model based on the fractal structure characteristics of fractures and pores. The model includes the influence of coal structure parameters, different original coal seam stress levels, and gas pressure on gas seepage characteristics.

In 1961, in order to completely reveal the nature of coal as a gas aggregate, Hodote of the former Soviet Union proposed the classification criteria of pores in coal: micropores (<10 nm), small pores (10-100 nm), mesopores (100-1000 nm), and macropores (>1000 nm) [26]. In 1985, the International Union of Pure and Applied Chemistry (IUPAC) proposed the classification scheme of pores in solid media as micropores (<2 nm), mesopores (2-50 nm), and macropores (>50 nm) [27]. The low temperature N_2_ adsorption method can test the pores with pore size of about 1.7 nm-300 nm, but it is not accurate enough to test the pores with pore size greater than 50 nm in practical application; the mercury intrusion porosimetry has a wide range of measurement, covering the pores of nanometer and micrometer scale, but mercury intrusion under high pressure is easy to cause damage to the pore structure, which leads to the inaccuracy of mercury porosimetry in the determination of pores with pore size less than 50 nm. Considering that the pores of coal adsorbing methane are mainly pores less than 50 nm (adsorption pores), and the pores greater than 50 nm are mostly seepage pores, the coal pores less than 10 nm are divided into micropores, 10-50 nm are divided into small pores, and greater than 50 nm are divided into macropores [28–30]. The quantitative or qualitative characterization methods of pores each have their own relatively precise scale ranges; combining multiple methods will make the results more convincing [31]. In order to ensure the comprehensive characterization of pore structure, the low temperature N_2_ adsorption method was used to characterize the micropores and small pores of coal samples, and the mercury intrusion porosimetry method was used to characterize the changes of macropores of coal samples. The combined application of the two methods is helpful to cross verify the evolution of pore structure caused by microwave irradiation cycles.

Although the pore structure of coal has been widely studied, the joint characterization of pore structure analysis results and fractal dimension evaluation, especially in different microwave irradiation cycles and between different coal ranks, is rarely reported. Fractal dimension analysis can provide a quantitative expression for the pore surface irregularity and pore size distribution heterogeneity, which is more conducive to understanding the micro level of the effect of microwave cycles on coal pore structure. In order to fill these gaps, the microwave irradiation cycles, pore structure, and fractal dimension of three coal ranks (subbituminous coal, bituminous coal, and anthracite coal) were jointly characterized in this study. In order to comprehensively understand the evolution process of pore structure of different ranks of coal under periodic microwave irradiation, the low-temperature N_2_ adsorption method, mercury intrusion porosimetry, and fractal theory were used.

Low permeability coal reservoirs pose a major challenge to efficient CBM recovery because traditional stimulation methods, such as hydraulic fracturing, often face many limitations, including water sensitivity, formation damage, and environmental concerns. Microwave irradiation provides an anhydrous and non-invasive stimulation means, which can induce thermal microfractures and enhance pore connectivity, so as to improve the permeability of coal and promote CBM desorption. However, the successful application of this technology requires a systematic understanding of how the microwave processing parameters, especially the number of irradiation cycles, affect the evolution of pore structure in different coal ranks. The purpose of this study is to meet this demand by clarifying the response of three typical coals to microwave irradiation cycles on the pore scale. This provides a scientific basis for optimizing intermittent microwave stimulation strategy to enhance the gas production capacity of low permeability CBM reservoir.

## Materials and methods

### Sample preparation

**Fig 1:**
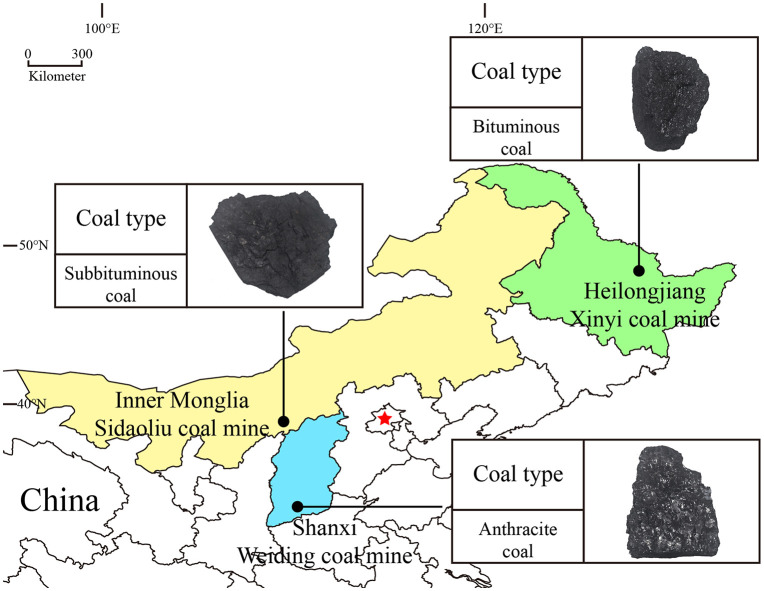
Collection of coal samples. The map was generated using public domain data from Natural Earth (http://www.naturalearthdata.com/).

The samples of subbituminous coal, bituminous coal, and anthracite prepared for the experiments were taken from the coal mines of Sidaoliu (Inner Mongolia, China), Xinyi (Heilongjiang, China), and Weiding (Shanxi, China), respectively, as shown in Fig 1. The coal seams at the sampling sites retained their native sedimentary structures, with intact bedding planes and only a small amount of secondary fissures developed. The selected samples were sealed with plastic film at the working face and sent to the laboratory as soon as possible to avoid changes in the chemical composition of coal caused by oxidation. The mean maximum vitrinite reflectance (Ro, max), the proximate analysis, and the ultimate analysis followed the standards of GB/T 6948-2008, GB/T 212-2008, and GB/T 476-2001, respectively. The analysis results are shown in Table 1.

**Table 1:** Petrologic characteristics, elemental composition, and proximate analysis of coal samples.

Samples	Ro, max (%)	Proximate analysis (wt. %)			Ultimate analysis (%, daf)				
		moisture^ad^	ash yield^ad^	volatile matter^daf^	C	H	O	N	S
Subbituminous coal	0.59	13.47	2.98	26.78	81.36	3.17	14.12	1.34	0.25
Bituminous coal	1.24	1.38	8.76	32.13	85.57	5.10	8.20	0.98	0.08
Anthracite coal	2.88	4.37	10.75	6.71	88.53	2.21	3.91	0.69	4.04

^ad^air dried basis.

^daf^dry ash-free basis.

### Experimental procedure

#### Microwave irradiation cycles.

Before the experiments, the coal samples were ground and sieved to obtain a size of 60-80 mesh for low-temperature N_2_ adsorption/desorption testing, and the cylindrical specimens with a size of φ 9×15 mm were obtained by drilling cores in the vertical bedding direction from the large coal samples for mercury intrusion testing.

**Fig 2:**
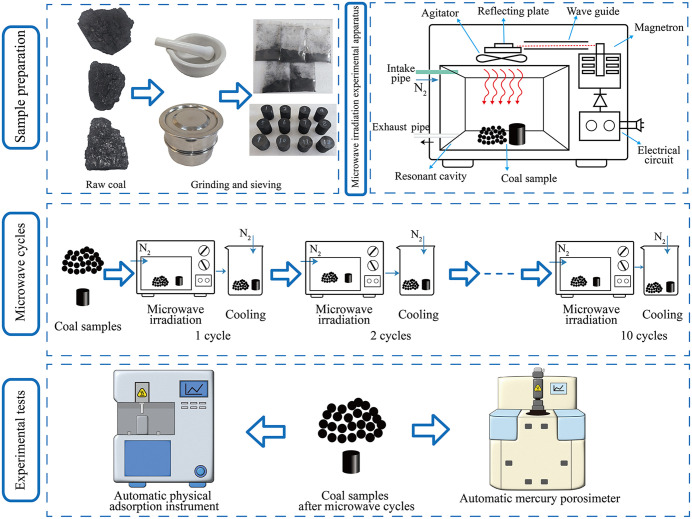
Sample preparation, Microwave cycles, and experimental tests.

The cyclic microwave modification experiment of the samples was carried out in a P70F20CL-DG(B0) microwave oven produced by Guangdong Galanz Company. The dimensions of the resonator in the oven were 180 mm high, 315 mm wide, and 329 mm deep. The frequency of the microwave oven was 2.45 GHz, and the power was 700 W. The sample preparation and testing equipment are shown in Fig 2.

The experimental process of microwave cycling is shown in Fig 2. The powder and cylindrical coal samples are placed in the resonant cavity of the microwave oven to be irradiated for 120 s at a power of 700 W, and then immediately moved to the beaker with N_2_ to cool to room temperature (to avoid coal sample oxidation). That is, a cycle is completed. According to this method, the microwave irradiation experiment was completed after 1, 3, 5, and 10 cycles. This choice is based on the range used in the previous study on Microwave cycle irradiation of coal (for example, 0, 1, 2, 3, 4 in Dong et al. [18] and 0, 2, 4, 6 in Qi et al. [19]), and it also extends the range to a higher number of cycles (10 cycles) to study the impact on coal beyond the previous number of cycles. This range also represents a gradual scheme of intermittent microwave stimulation in practical application, which can systematically evaluate the evolution of pore structure with the increase of cumulative microwave irradiation.

#### Low temperature N_2_ Adsorption/Desorption Test (LT-N_2_GA).

The low temperature N_2_ adsorption/desorption tests were carried out with a Micromeritics ASAP 2020 Plus Automatic physical adsorption instrument produced by Micromeritics company, in accordance with the standards of ISO 15901-2-2006 and ISO 15901-3-2007.

The key to testing and reflecting the pore structure of coal using low temperature N_2_ adsorption method lies in two points: firstly, it is necessary to accurately testing the N_2_ adsorption/desorption isotherm of the coal samples; secondly, it is necessary to select an appropriate model to calculate the pore volume, specific surface area, and other pore structure parameters of the coal samples [32,33].

This test conducts N_2_ adsorption at a temperature of 77.4 K. After the completion of adsorption, the N_2_ adsorption/desorption isotherm of the coal samples within the relative pressure P/P_0_ range of 0.01 to 0.99 is obtained. “P” is the adsorption equilibrium pressure, and “P_0_” is the saturated vapor pressure.

Due to the formation of adsorption liquid films during single-layer and multi-layer adsorption of N_2_ in the adsorption process, the thickness of the liquid film and N_2_ pressure are related to the surface properties of coal samples, and can be calculated based on Halsey’s equation.


$$\begin{array}{c} d_{L}=\text{0}.\text{354}{[-\text{5}/\text{ln}\left(P/P_{\text{0}}\right))]}^{\text{1}/\text{3}} \end{array}$$ (1)

In the formula, $d_{L}$ is the thickness of the liquid film, in nanometers (nm).

According to the Kelvin equation, the relationship between the relative pressure of N_2_ and the curvature of N_2_ condensation in the pore can be calculated [34]:


$$\begin{array}{c} r_{k}=-\frac{\text{2}\gamma_{L}V_{Lmol}}{RT_{b}\text{ln}\left(P/P_{\text{0}}\right)}=-\frac{\text{0}.\text{953}}{\text{ln}\left(P/P_{\text{0}}\right)} \end{array}$$ (2)

In the formula, ${\text{r}}_{\text{k}}$ is the curvature radius of N_2_ gas condensed in the pore, in nanometers (nm); $\gamma_{\text{L}}$ is the surface tension of liquid condensate, 0.008876 N/m; $V_{Lmol}$ is the molar volume of liquid condensate, 0.034752 L/mol; *R* is the universal gas constant 8.314 J/(mol·K); $T_{b}$ is the test temperature, 77.4K; Then the corrected actual radius $r_{p}$ of the pore is:


$$\begin{array}{c} r_{p}=r_{k}+t \end{array}$$ (3)

The pore size can be calculated according to the measured N_2_ adsorption quantity and relative pressure data; then, the pore volume and specific surface area of the coal sample micropores (d<10 nm) and small pores (10 nm<d<50 nm) can be further calculated by pore size.

#### Mercury intrusion test.

The mercury intrusion test uses the PoreMaster 33G automatic Mercury Porosimeter produced by Quantachrome, USA. The coal sample size is φ 9×10 mm, the mercury injection pressure range is 0-33000 psi, and the corresponding pore size range is 6.4-263900 nm.

In 1805, Laplace derived that the work done in expanding a non-spherical liquid surface with principal curvatures R_1_ and R_2_ is equal to the work acting on the concave surface of the liquid:


$$\begin{array}{c} \Delta P=\gamma (\frac{\text{1}}{R_{\text{1}}}+\frac{\text{1}}{R_{\text{2}}}) \end{array}$$ (4)

In the formula, ΔP is the pressure on the liquid level, MPa; γ is the surface tension of the liquid, mN/m.

Assuming the pore is cylindrical, based on Laplace, Washburn [35–37] obtained:


$$\begin{array}{c} r_{Hg}=-\frac{\text{2}\sigma_{H_{g}}\text{cos}\theta_{H_{g}}}{P_{Hg}} \end{array}$$ (5)

In the formula, $r_{Hg}$ is the pore radius, μm; $P_{Hg}\;$ is the absolute mercury inlet pressure, MPa; $\theta_{H_{g}}$ is the contact angle between mercury and pore surface, generally 140°, $\sigma_{H_{g}}$ is the interfacial tension of mercury, 0.48 J/m^2^.Then formula (5) is:


$$\begin{array}{c} r_{Hg}=\frac{\text{0}.\text{735}}{P_{Hg}} \end{array}$$ (6)

The above equation is the basic principle of measuring aperture by the mercury intrusion method [38,39]. When the pressure changes from $P_{Hg\text{1}}$ to $P_{Hg\text{2}}$, corresponding to the pore sizes $r_{Hg\text{1}}$ and $r_{Hg\text{2}}$, respectively. Using a mercury porosimeter to measure the pore volume of unit mass coal samples pressed in two pore sizes, the pore size distribution can be obtained. The specific surface area distribution can be derived from the pore size distribution.

### Fractal model

#### FHH fractal.

The Frenkel-Halsey-Hill (FHH) fractal model is the main method for calculating the fractal dimension of coal micropores using low temperature N_2_ adsorption, both domestically and internationally. It is a relatively authoritative model used to reflect the irregularity of pore surface and pore size distribution in coal samples, calculated based on low temperature N_2_ adsorption experimental data [40].

The calculation formula for the fractal FHH model is [41]:


$$\begin{array}{c} \text{ln}\left(\frac{V}{V_{\text{0}}}\right)=\text{A}\text{ln}\left[\text{ln}\frac{P}{P_{\text{0}}}\right]+{\text{C}}_{\text{1}} \end{array}$$ (7)

It can also be written as:


$$\begin{array}{c} \text{ln}V=\text{A}\text{ln}\left[\text{ln}\frac{P}{P_{\text{0}}}\right]+{\text{C}}_{\text{2}} \end{array}$$ (8)

In the formula, *V* is the adsorption capacity at N_2_ pressure P,cm^3^/g; $V_{\text{0}}$ is the adsorption capacity of N_2_ single-layer adsorption, cm^3^/g; $P_{\text{0}}$ is N_2_ saturation adsorption pressure, MPa; *P* is N_2_ adsorption pressure, MPa; ${\text{C}}_{\text{1}}$ and ${\text{C}}_{\text{2}}$ are fitting constants, dimensionless; A is the curve slope obtained by FHH fitting with $\text{ln}\left[\text{ln}P/P_{\text{0}}\right]$ as the independent variable and lnV as the dependent variable.

The fractal dimension of pore surface and pore size distribution of micropores and small pores in coal samples is [42]:


$$\begin{array}{c} \text{D}=\text{A}+\text{3} \end{array}$$ (9)

The FHH fractal curves can be divided into two parts with P/P_0_=0.5 as the boundary [43]. When 0<P/P_0_<0.5, the fractal dimension D_1_ is used to characterize the surface irregularity of micropores and small pores in coal samples; When 0.5<P/P_0_<1, the fractal dimension D_2_ is used to characterize the irregularity and pore size distribution of micropores and small pores.

#### Menger fractal.

The Menger sponge model is based on mercury intrusion test data to characterize the fractal dimension D of macropore size distribution in coal samples [44,45]. According to the relationship between pore size distribution ${dV}_{cum}/{dr}_{Hg}$ and fractal dimension D:


$$\begin{array}{c} \frac{{dV}_{cum}}{{dr}_{Hg}}=k_{\text{1}}r_{Hg}^{\text{2}-D} \end{array}$$ (10)

In the formula, $V_{cum}$ is the cumulative mercury injection amount, approximately equal to the cumulative pore volume, cm^3^/g; $k_{\text{1}}$ is a proportionality constant, dimensionless.

Combined with formula (5) Washburn equation, it can be concluded that:


$$\begin{array}{c} \frac{{dV}_{cum}}{{dP}_{Hg}}=k_{\text{2}}P_{Hg}^{D-\text{4}} \end{array}$$ (11)

In the formula, $k_{\text{2}}$ is the proportional constant, dimensionless. By taking the logarithm on both sides, it can be concluded that:


$$\begin{array}{c} \text{log}(\frac{{dV}_{cum}}{{dP}_{Hg}})=\text{log}(k_{\text{2}})+(\text{D}-\text{4})\text{log}\left(P_{Hg}\right) \end{array}$$ (12)

According to the above formula, the slope of $\text{log}{dV}_{cum}/{dP}_{Hg}$—$\text{log}\left(P_{Hg}\right)$ curves can be obtained by fitting the mercury injection curves data, so as to obtain the fractal dimension D of macropore diameter distribution.

## Results and analysis

### Low temperature N_2_ adsorption/desorption curve of coal samples

**Fig 3:**
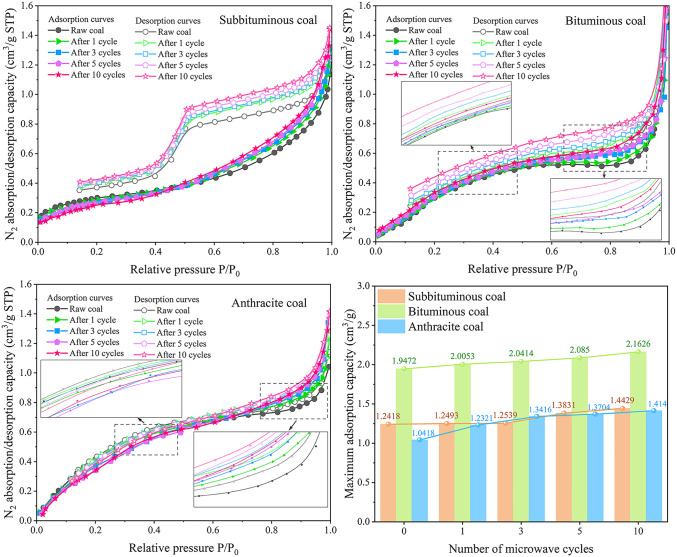
The N_2_ adsorption/desorption curves of coal samples with microwave irradiation cycles.

Fig 3 shows the low temperature N_2_ adsorption/desorption isotherms of coal samples under microwave irradiation for 1, 3, 5, and 10 cycles. According to the classification standard of IUPAC adsorption isotherms, all adsorption isotherms of coal samples before and after microwave irradiation cycles are close to type IV [30].

The N_2_ adsorption process is divided into three stages. When the relative pressure P/P_0_<0.4, the curves protrude upward, indicating that the main form of N_2_ adsorption is single-layer adsorption, and the adsorption isotherm rises slowly. When the relative pressure 0.4<P/P_0_<0.9, the adsorption form of N_2_ changed from single-layer adsorption to multi-layer adsorption, and then capillary condensation occurred, and the adsorption isotherm rises rapidly. When the relative pressure P/P_0_ approaches 1, the adsorption curve rises sharply. The main reason is that there are pores larger than 300 nm in the coal samples, and when the relative pressure is close to the saturated vapor pressure, it is difficult for N_2_ to reach adsorption saturation in these pores. The shape of adsorption/desorption isotherms of three kinds of coal samples did not change significantly after microwave irradiation cycles, but the amount of adsorption/desorption changed, indicating that microwave irradiation cycles did not change the overall type of pores of coal samples, but mainly changed the pore volume distribution of coal samples.

For the subbituminous coal sample, the desorption isotherm and adsorption isotherm did not coincide to form a hysteresis loop, which can reflect the pore type of the coal sample to a certain extent. The hysteresis loops of all coal samples before and after microwave irradiation cycles are close to H_3_ type, and the hysteresis loops are large, indicating that there are more narrow slit pores in the coal samples [46]. The maximum adsorption capacity of the natural coal sample of subbituminous samples is 1.2418 cm^3^/g, and the maximum adsorption capacity of N_2_ increases to 1.2493, 1.2539, 1.3831, and 1.4429 cm^3^/g after microwave irradiation for 1, 3, 5, and 10 cycles, respectively, indicating that microwave irradiation cycles increase the total pore volume of subbituminous coal samples.

For the bituminous coal sample, the hysteresis loop is not obvious, which is similar to the H_3_ type. When the relative pressure P/P_0_>0.5, the difference between the adsorption branch and desorption branch appears, indicating that there are wider slit pores in the coal samples. The maximum adsorption capacity of the natural coal sample of bituminous coal samples is 1.9472 cm^3^/g, and the maximum adsorption capacity of N_2_ increases to 2.0053, 2.0414, 2.0850, and 2.1626 cm^3^/g after microwave irradiation for 1, 3, 5, and 10 cycles, respectively, indicating that microwave irradiation cycles increase the total pore volume of bituminous coal samples.

For the anthracite coal sample, the hysteresis loop is small and similar to the H_3_ type. This indicates that there are cylindrical pores and semi-open pores in the coal samples. The maximum adsorption capacity of the natural coal sample of anthracite samples is 1.0418 cm^3^/g, and the maximum adsorption capacity of N_2_ increases to 1.2321, 1.3416, 1.3704, and 1.4140 cm^3^/g after microwave irradiation for 1, 3, 5, and 10 cycles, respectively, indicating that microwave irradiation cycles increase the total pore volume of anthracite coal samples.

### Pore volume and specific surface area of micropores and small pores in coal samples

**Fig 4:**
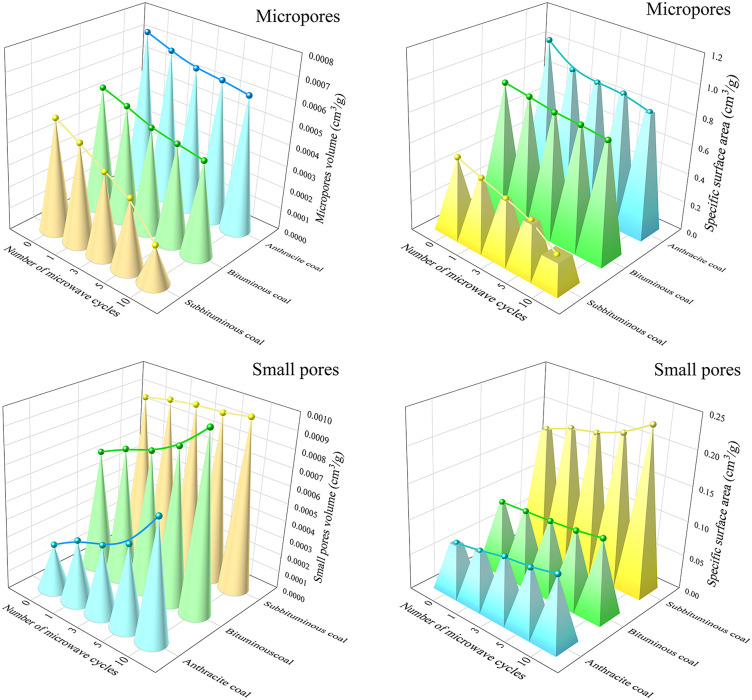
The variation of the volume and specific surface area of micropores and small pores in the three kinds of coal samples with the number of microwave irradiation cycles.

Fig 4 shows the variation curves of the volume and specific surface area of micropores and small pores in the three kinds of coal samples with the number of microwave irradiation cycles. It can be seen that the micropore volume and specific surface area of the three kinds of coal samples decrease with the increase in microwave irradiation cycles. For small pores, the results show that with the increase of microwave irradiation cycles, the pore volume and specific surface area of the three kinds of coal samples showed a significant increasing trend.

After microwave irradiation cycles, the pore volume and specific surface area of micropores decreased, and the pore volume and specific surface area of small pores increased. Under the action of microwave radiation, some micropores gradually expand and connect into small pores, resulting in the reduction of micropores and the increase of small pores. The reason is that bound water in coal and bound water in minerals are evaporated and removed, and high-pressure steam promotes pore expansion and connectivity. Part of methyl and methylene on the side chain of coal molecular alkanes break after microwave irradiation, and volatilize in the form of small molecular organics, which will also promote the development and expansion of pores.

### FHH fractal dimensions of coal samples

**Fig 5:**
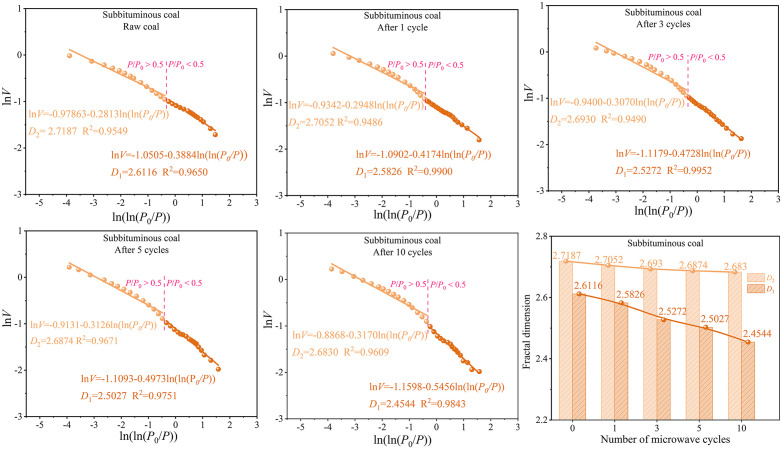
FHH fractal dimensions of subbituminous coal sample with the number of microwave irradiation cycles.

**Fig 6:**
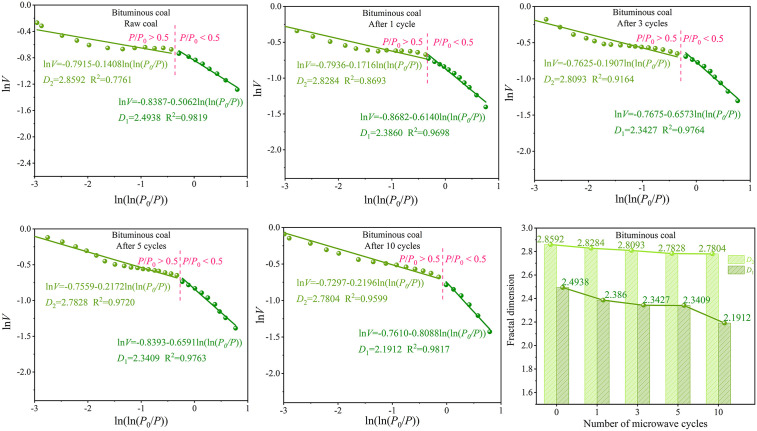
FHH fractal dimensions of the bituminous coal sample with the number of microwave irradiation cycles.

**Fig 7:**
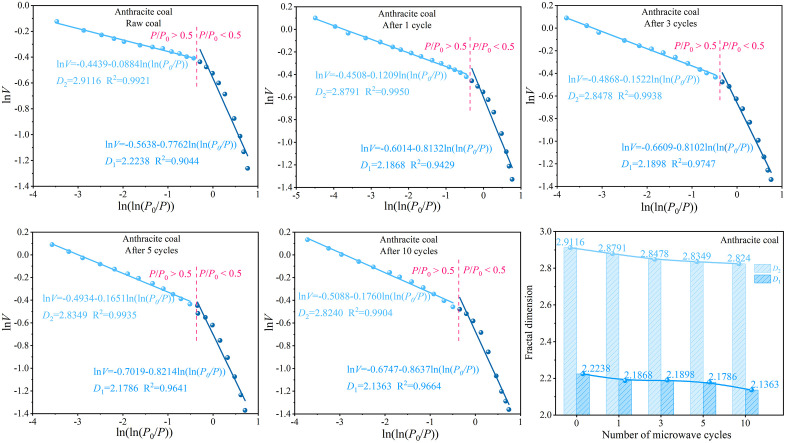
FHH fractal dimensions of the anthracite coal sample with the number of microwave irradiation cycles.

Fig 5, Fig 6, Fig 7 show the calculation results of D_1_ and D_2_ of three kinds of natural coal samples and coal samples after microwave irradiation for 1, 3, 5, and 10 cycles. It can be seen that the values of D_1_ and D_2_ of the three kinds of coal samples before and after the irradiation cycles are between 2 and 3, and the surface irregularity and pore size distribution irregularity of the coal samples before and after microwave irradiation cycles have good fractal characteristics [47].

As shown in the figure, for the raw coal samples, the irregularity of micropores and small pores surfaces follows the order: subbituminous coal sample>bituminous coal sample>anthracite coal sample. After 10 microwave irradiation cycles, D_1_ gradually decreases, indicating a reduction in the surface irregularity of micropores and small pores across all coal samples. The degree of reduction follows the order: bituminous coal sample>subbituminous coal sample>anthracite coal sample, with decreases of 12.13%, 6.02%, and 3.93%, respectively. Regarding the irregularity of pore size distribution in micropores and small pores, the raw coal samples show the following order: anthracite coal sample>bituminous coal sample>subbituminous coal sample. After 10 microwave irradiation cycles, D_2_ slowly decreases, indicating a reduction in the irregularity of pore size distribution for micropores and small pores. The degree of reduction follows the order: anthracite coal sample>bituminous coal sample>subbituminous coal sample, with decreases of 3.01%, 2.76%, and 1.31%, respectively.

After 1, 3, 5, and 10 microwave irradiation cycles, all three kinds of coal, D_1_ and D_2_, showed a downward trend. This indicates that the surface irregularity of micropores and small pores, as well as the irregularity of pore size distribution, in the three kinds of coal samples decreased after cycling and decreased with increasing number of cycles. For subbituminous coal, compared with D_1_, the change of D_2_ is not obvious, which indicates that the reduction of the irregularity of micropores and pore size is less than that of the surface irregularity. For bituminous coal and anthracite coal, both D_1_ and D_2_ show significant reductions.

The reason for the decrease of fractal dimensions D_1_ and D_2_ of coal samples after microwave irradiation cycles is that, on the one hand, the pore thinning effect formed by the evaporation of bound water and mineral bound water in coal under microwave radiation makes the surface of micropores and small pores of coal sample more flat, and on the other hand, the expansion and connectivity of pores under microwave radiation slightly improves the order of micropores arrangement.

### Mercury intrusion-extrusion curves of coal samples

**Fig 8:**
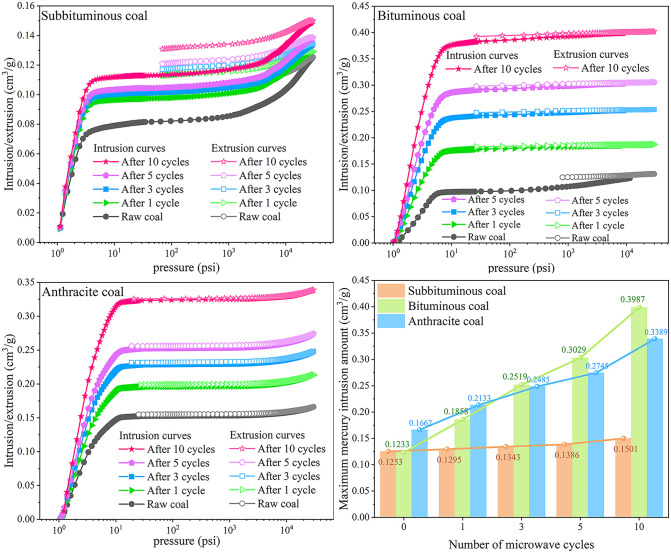
Effect of microwave irradiation cycles on the mercury intrusion curves and maximum mercury intrusion amounts of three types of coal.

Fig 8 shows the mercury intrusion-extrusion curves of three kinds of coal samples after 1, 3, 5, and 10 microwave irradiation cycles. It can be seen that the mercury withdrawal curves all exhibit a “hysteresis phenomenon”, that is, the mercury withdrawal curves do not coincide with the mercury entry curves [48].

The results showed that with the increase of microwave irradiation cycles, the maximum mercury intrusion rate of the three coal samples all showed a growing trend, and the degree of increase was: bituminous coal>anthracite coal>subbituminous coal.

### Pore volume and specific surface area of macropores in coal samples

**Fig 9:**
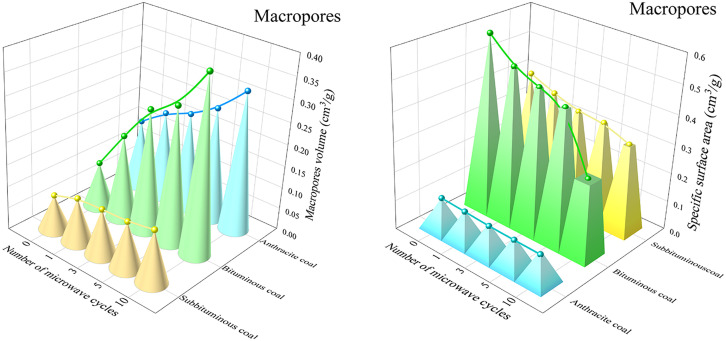
Change of pore volume and specific surface area of macropores in coal samples with the number of microwave irradiation cycles.

Fig 9 shows the variation curve of macropore volume and specific surface area of three kinds of coal samples with the number of microwave irradiation cycles. From the above, it can be seen that as the number of microwave irradiation cycles increases, the pore volume of the three kinds of coal samples’ macropores increases to varying degrees, while the specific surface area of the macropores decreases to varying degrees. Among natural coal samples, the anthracite coal sample has the largest macropore volume, followed by the bituminous coal sample, and the subbituminous coal sample has the smallest. The specific surface area of the macropores in the bituminous coal sample is the largest, followed by the subbituminous coal sample, and the anthracite coal sample is the smallest.

The reason for the increase of the macropores volume and the decrease of the specific surface area of the coal sample is that on the one hand, the pore expansion effect of the high-pressure steam generated by the evaporation of water in the coal promotes the connectivity of the macropores; on the other hand, the heterogeneous thermal stress formed at the interface between coal matrix and mineral under microwave irradiation cycles promotes the development and expansion of macropores.

### Menger fractal dimensions of coal samples

**Fig 10:**
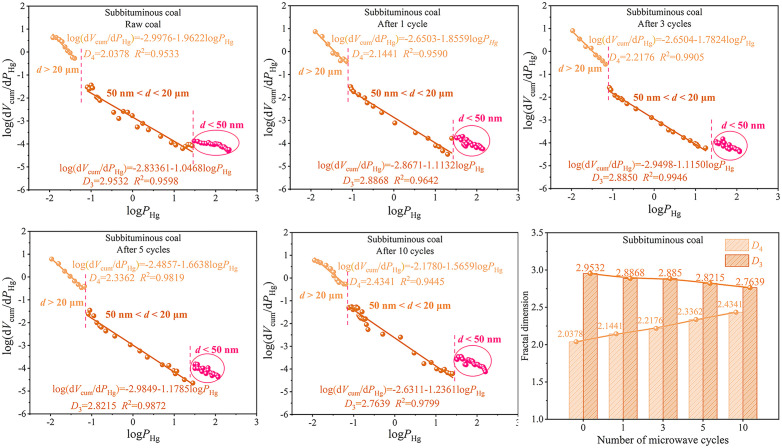
Menger fractal dimensions of the subbituminous coal sample with the number of microwave irradiation cycles.

**Fig 11:**
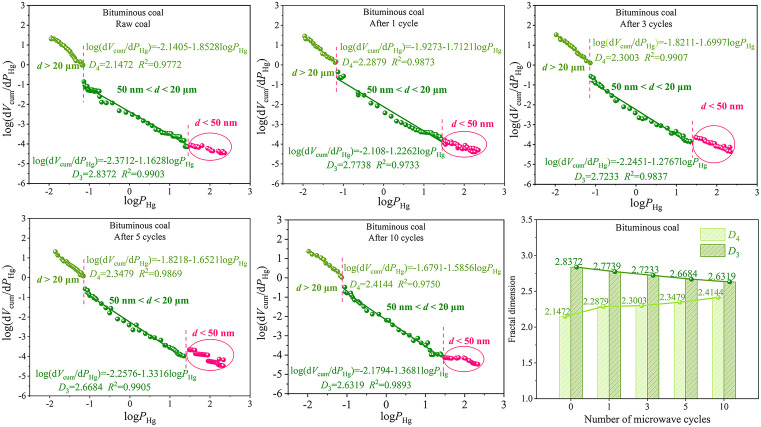
Menger fractal dimensions of the bituminous coal sample with the number of microwave irradiation cycles.

**Fig 12:**
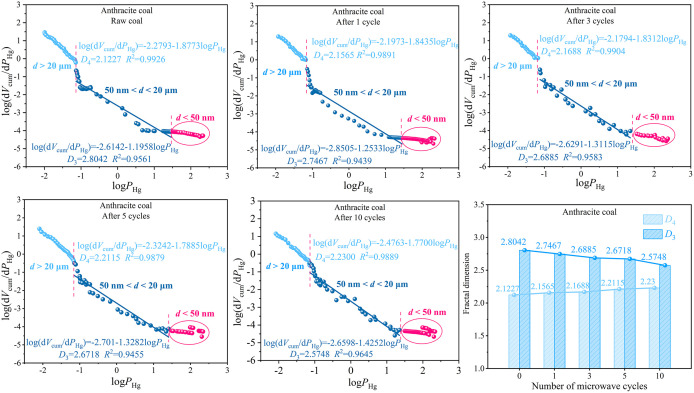
Menger fractal dimensions of the anthracite coal sample with the number of microwave irradiation cycles.

Fig 10, Fig 11, Fig 12 show the change in the fractal dimension of the macropore size distribution irregularity of three kinds of coal samples before and after microwave irradiation cycles. It can be seen that the fractal dimension fitting curve of macropore diameter distribution of three kinds of coal samples has a similar trend compared with the natural coal samples and the coal samples after 1, 3, 5, and 10 microwave irradiation cycles. All fractal curves are divided into three sections. The fractal dimension D3 (light part) represents the irregularity of macropores with an pore size of 50 nm-20 μm; The fractal dimension D4 (dark part) represents the irregularity of the distribution of macropores with an aperture greater than 20 μm. The pink part represents pore sizes smaller than 50 nm.

From the above, it can be seen that for macropores with pore sizes ranging from 50 nm to 20 μm, the irregularity of pore size distribution in natural coal samples is as follows: subbituminous coal sample>bituminous coal sample>anthracite coal sample. After 10 irradiation cycles of microwave, the irregularity of pore size distribution decreased, and the degree of reduction was as follows: anthracite sample>bituminous coal sample>subbituminous coal sample, with reductions of 8.18%, 7.24%, and 6.41%, respectively. For macropores with a pore size greater than 20 μm, the irregularity of pore size distribution in natural coal samples is as follows: anthracite coal sample>bituminous coal sample>subbituminous coal sample. After 10 microwave irradiation cycles, the irregularity of pore size distribution increased, and the degree of increase was as follows: subbituminous coal sample>bituminous coal sample>anthracite coal sample, with increases of 19.4%, 12.4%, and 5.05%, respectively.

After microwave irradiation cycles, the irregularity of macropores size distribution of coal samples with pore size of 50 nm-20 μm decreases, while the irregularity of macropores size distribution with pore size greater than 20 μm increases. This phenomenon is attributed to two aspects: on the one hand, the pore heterogeneity is reduced and the pore arrangement is more uniform and orderly due to the pore enlargement and pore thinning effect of microwave irradiation cycles; On the other hand, the heterogeneous thermal stress generated in the coal sample under microwave radiation leads to the tearing damage of the coal sample, giving birth to some irregular macropores. The former plays a leading role for macropores with a pore size of 50 nm-20 μm, while the latter plays a leading role for macropores with a pore size of more than 20 μm.

### Pore size distribution

**Fig 13:**
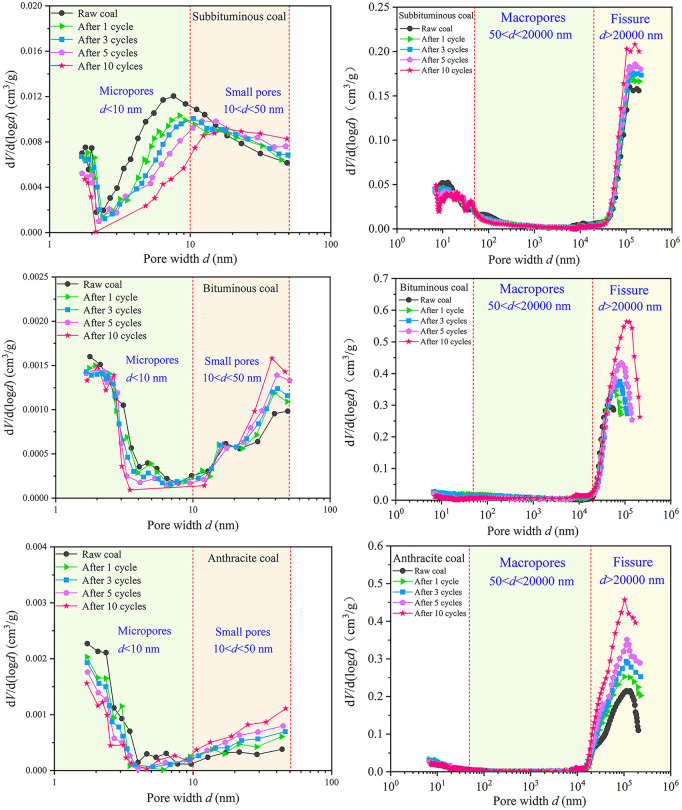
Effect of microwave irradiation cycles on the pore size distribution of micropores, small pores, macropores, and fissures.

Fig 13 shows the pore size distribution of micropores, small pores, macropores, and fissures before and after microwave irradiation cycles. From the Fig 13, it can be seen that as the number of cycles increases, the number of micropores gradually decreases while the number of small pores gradually increases. This indicates that microwaves increase the pore size of micropores and transform them into small pores. For macropores, the change is not very significant, but for fissures with a pore size greater than 20000 nm, their number increases significantly after microwave irradiation cycles, and it is most obvious at 10^5^ nm.

### Microwave irradiation mechanism

**Fig 14:**
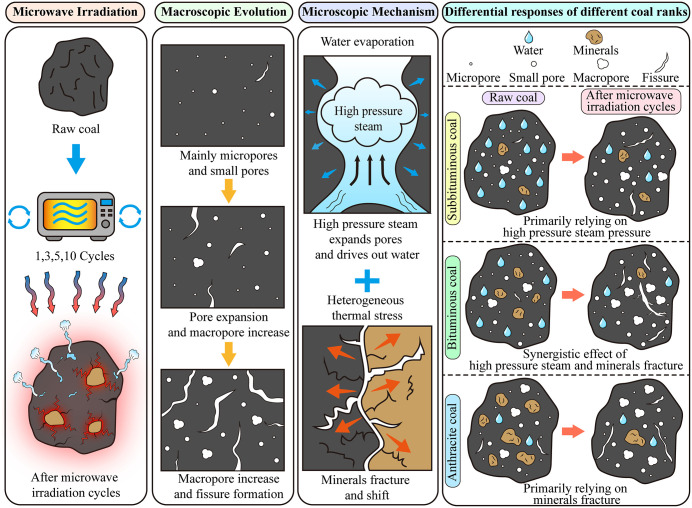
Schematic diagram of microwave irradiation modification principle.

Compared with the traditional pyrolysis process, microwave irradiation technology provides volumetric and selective heating functions, which can realize the transformation of pore structure faster and more energy-saving. Different from chemical methods (such as acid/alkali treatment), microwave treatment is an anhydrous and reagent free treatment method, which can avoid secondary pollution. In order to clarify the mechanism behind microwave-induced pore structure evolution, the modification principle is shown in Fig 14. As shown in the figure, the microwave irradiation cycle has changed the pore structure and connectivity of coal from two aspects. One is that the internal bound water of coal is transformed into high-pressure steam by microwave; The second is that microwave causes thermal stress in the coal, leading to mineral fracture and pore increase.

The differences in the responses of coals of different ranks to microwave irradiation cycles can be further explained by combining their basic properties with the evolution of pore structure and fractal characteristics. Subbituminous coal has the highest moisture content (13.47%) and oxygen content (14.12%). After 10 cycles, its maximum mercury intrusion amount increases only from 0.1253 to 0.1501 cm^3^/g, which is the smallest increase among the three coals, whereas D_4_ shows the most pronounced increase. This indicates that the response of subbituminous coal is more closely related to pore expansion caused by steam pressure. It is mainly manifested by the transformation of micropores into small pores, accompanied by limited fissures extension, while the overall growth of macropores remains relatively weak. Bituminous coal shows the strongest overall response. Its maximum mercury intrusion amount increases from 0.1233 to 0.3987 cm^3^/g, and D_1_ exhibits the largest decrease, suggesting the most evident smoothing of pore surfaces, development of macropores, and enhancement of fissures connectivity. This implies a stronger combined effect of steam pressure expansion and heterogeneous thermal stress. Anthracite has the lowest volatile matter content (6.71%) and oxygen content (3.91%), but a relatively high ash yield (10.75%). Its maximum mercury intrusion amount increases from 0.1662 to 0.3389 cm^3^/g, and the decrease in D_3_ is the greatest, while the increase in D_4_ is relatively limited. These results suggest that anthracite is characterized more by redistribution of pore sizes and thermal stress induced reorganization of macropores and fissures within a dense matrix, in which thermal mismatch between minerals and the coal matrix may play a more important role.

Microwave irradiation cycles alter the pore and fracture structure and connectivity of coal, expanding some micropores into small pores, increasing the number of small pores, and enhancing the connectivity of both macropores and fissures. This leads to an increase in the pore volume of coal. It should be noted that this study selected only one representative sample each of subbituminous coal, bituminous coal, and anthracite coal for analysis; therefore, the possible differences among samples of the same coal rank under microwave irradiation cycles were not further examined. In addition, under fixed irradiation conditions of 120 s and 700 W, this work mainly focused on the effect of the number of irradiation cycles on coal pore structure evolution. Since microwave irradiation time and output power are also important factors influencing the modification effect, future studies should further investigate the synergistic effects of irradiation duration, microwave power, and cycle number on the evolution of coal pore structures. The study reveals the effect of microwave irradiation cycles on the pore structure and fractal dimension of coal, aiming to provide a theoretical basis and technical support for improving the permeability of low-permeability coal seams and enhancing the extraction efficiency of CBM through efficient microwave cycles.

## Conclusion

(1) Microwave irradiation cycles do not change the overall type of pores in coal samples, but only change the pore volume distribution of coal samples.
(2) For micropores and small pores, with the increase of microwave irradiation cycles, the micropore volume and specific surface area of the three kinds of coal samples decrease, while the small pore volume and specific surface area increase. The FHH fractal dimension calculation results show that the microwave cycle reduces the irregularity of micropores and small pore surface and pore size distribution of coal samples.
(3) For macropores, with the increase in the number of microwave irradiation cycles, the macropore volume of the three kinds of coal samples increases, and the specific surface area of macropores decreases. The Menger fractal dimension calculation results show that the microwave irradiation cycles reduce the irregularity of the macropore size distribution of the coal samples with a pore size of 50 nm-20 μm, and increase the irregularity of the macropore size distribution with a pore size greater than 20 μm.
(4) Microwave irradiation cycles promote the development, expansion, and connectivity of pores and fractures, make some micropores expand into small pores, increase the number of small pores, and enhance the connectivity of macroporous fissures. The main reasons are as follows: the bound water in coal and the bound water in minerals are evaporated and removed under microwave radiation, and the high-pressure steam formed has a certain effect of pore expansion and drainage; The heterogeneous thermal stress formed at the interface between coal matrix and minerals under microwave radiation makes some minerals fracture and shift.
(5) From the perspective of low-permeability CBM reservoir stimulation, microwave irradiation cycles reconstruct the multiscale pore–fracture system of coal by promoting the transformation of micropores into small pores, increasing the development of macropores, and enhancing the connectivity of macroporous fissures. These structural changes may facilitate the formation of more continuous gas transport pathways within coal and provide a pore-structure basis for improving CBM migration under microwave cyclic modification.

## Supporting information

S1 Data Minimal data set. (ZIP)

## References

[1] Jermain DO, Pilcher RC, Ren ZJ, Berardi EJ. Coal in the 21st century: Industry transformation and transition justice in the phaseout of coal-as-fuel and the phase-in of coal as multi-asset resource platforms. Energy Clim Chang. 2024;5:100142. doi: 10.1016/j.egycc.2024.100142

[2] Wang H, Shen J, Gao J, Wang W, Zhu L, Gu Y, et al. Cost estimation of Non-CO2 greenhouse gas emissions reduction- a bottom-up analysis of coal-bed methane extraction and utilization in Shanxi, China. Energy. 2024;309:133007. doi: 10.1016/j.energy.2024.133007

[3] Li G, Zhang J, Zhao Q, Chen H, Chen Y, Zhang G. Evaluation methodology of coal-rock gas resources and exploration potential of favorable areas in onshore China. Pet Explor Dev. 2025;52(6):1389–406. doi: 10.11698/PED.20250535

[4] Chen S, Tao S, Tang D. In situ coal permeability and favorable development methods for coalbed methane (CBM) extraction in China: From real data. Int J Coal Geol. 2024;284:104472. doi: 10.1016/j.coal.2024.104472

[5] Leng K, Guan B, Liu W, Jiang C, Cong S, Xin Y. Research progress of coalbed methane extraction. Energy Rep. 2024;12:5728–46. doi: 10.1016/j.egyr.2024.11.030

[6] Wang K, Guo L, Xu C, Wang W, Yang T, Lin S, et al. Multiscale characteristics of pore-fracture structures in coal reservoirs and their influence on coalbed methane (CBM) transport: A review. Geoenergy Sci Eng. 2024;242:213181. doi: 10.1016/j.geoen.2024.213181

[7] Peng X, Ji H. Control mechanism of small organic molecules on methane adsorption capacity of coal. Fuel. 2023;331:125904. doi: 10.1016/j.fuel.2022.125904

[8] Gao D, Liang J, Hong L, Zheng D, Yang Z, Wang J. Effect of small-molecule organic matter on methane adsorption in anthracite. J Saudi Chem Soc. 2024;28(6):101930. doi: 10.1016/j.jscs.2024.101930

[9] Liang X, Kang T, Kang J, Zhang X, Zhang L, Li H, et al. Experimental study of influence of natural organic solvent limonene on methane adsorption–desorption behaviors of selected rank coals. Energy. 2024;291:130491. doi: 10.1016/j.energy.2024.130491

[10] Wang F, Zhang X, Zhang S, Wang K. Mechanism of solvent extraction-induced changes to nanoscale pores of coal before and after acidification. Fuel. 2022;310:122467. doi: 10.1016/j.fuel.2021.122467

[11] Chang Y, Yao Y, Liu D, Liu Y, Cui C, Wu H. Behavior and mechanism of water imbibition and its influence on gas permeability during hydro-fracturing of a coalbed methane reservoir. J Pet Sci Eng. 2022;208:109745. doi: 10.1016/j.petrol.2021.109745

[12] Zhu Y, Li C, Cao H, Wu L, Yang F, Fu S, et al. Effects of spatial distribution of tar-rich coal and oil shale and primary factors on product characteristics during microwave co-pyrolysis. Fuel. 2025;385:134085. doi: 10.1016/j.fuel.2024.134085

[13] Liu B, Gu X, Li Z, Yang B, Wang H, Liu J. Exploring microwave activation as a novel method for activating coal gangue: Focus on microwave activation mechanisms and hydration characteristics of cementitious supplementary materials. Constr Build Mater. 2024;450:138482. doi: 10.1016/j.conbuildmat.2024.138482

[14] Tang L, Chen X, Tao X. Non-thermal effect of microwave on organic sulfur removal from coal by microwave with peroxyacetic acid. Fuel. 2023;338:127262. doi: 10.1016/j.fuel.2022.127262

[15] Li J, Zhang J, Xiao Q, Liu B, Lin W, Li W. Experimental study on differential thermal response and pore-fracture structure evolution characteristics of coals under microwave irradiation: a case study of five different rank coals. Energy Fuels. 2024;38(11):9497–514. doi: 10.1021/acs.energyfuels.4c01228

[16] Yang Z, Wang C, Zhao Y, Bi J. Microwave fracturing of frozen coal with different water content: Pore-structure evolution and temperature characteristics. Energy. 2024;294:130938. doi: 10.1016/j.energy.2024.130938

[17] Lu J, Zheng C, Liu W, Li H, Shi S, Lu Y, et al. Evolution of the pore structure and fractal characteristics of coal under microwave-assisted acidification. Fuel. 2023;347:128500. doi: 10.1016/j.fuel.2023.128500

[18] Dong M, Feng L, Qin B. Characteristics of coal gasification with CO2 after microwave irradiation based on TGA, FTIR and DFT theory. Energy. 2023;267:126619. doi: 10.1016/j.energy.2023.126619

[19] Qi X, Liu X, Ma H, Liu Z, Xie W, Zhang Y, et al. Study on the damage and seepage characteristics of water-saturated coal by microwave cycling. Sci Rep. 2024;14(1):17514. doi: 10.1038/s41598-024-68506-4 39079953 PMC11289454

[20] Huang X, Ning X, Mao J, Li P, Ren J, Yang Q, et al. Comparative study on microwave and conventional pyrolysis characteristics of Tar-Rich coal. Fuel. 2026;405:136790. doi: 10.1016/j.fuel.2025.136790

[21] Chemerinskiy M, Kuzmin A, Pinchuk V, Pinchuk S. Microwave-induced alterations in the structure of coals at different metamorphic stages. Fuel. 2025;381:133326. doi: 10.1016/j.fuel.2024.133326

[22] Zhu Y, Liu H, Wang T, Wang Y, Liu H. Evolution of pore structures and fractal characteristics of coal-based activated carbon in steam activation based on nitrogen adsorption method. Powder Technol. 2023;424:118522. doi: 10.1016/j.powtec.2023.118522

[23] Wang L, Cheng L, Yin S, Chen W, Li H, Li S, et al. Pore structure evolution and fractal characteristics of sandstone uranium ore under different leaching temperatures. Powder Technol. 2025;454:120713. doi: 10.1016/j.powtec.2025.120713

[24] Zha W, Lin B, Liu T, Liu T, Yang W, Zhang X. Effect of the pore structure characteristics of coal samples on the dynamic adsorption of water vapor. Fuel. 2024;366:131365. doi: 10.1016/j.fuel.2024.131365

[25] Liu Z, Han J, Yang H, Lv J, Dong S. A new model for coal gas seepage based on fracture-pore fractal structure characteristics. Int J Rock Mech Min Sci. 2024;173:105626. doi: 10.1016/j.ijrmms.2023.105626

[26] Shao X, Qin B, Shi Q, Li Z, Qu B, Xu S, et al. Influence mechanism of pore structure evolution on oxygen consumption dynamics during low-temperature oxidation of igneous metamorphic coal. Int J Min Sci Technol. 2026;36(3). doi: 10.1016/j.ijmst.2026.01.001

[27] Liu Y, Zhang Y, Gao X, Cheng Y, Wang S. CO2 adsorption equilibrium model incorporating pressure-dependent multiscale pore fractal evolution in coal. Energy. 2025;337:138640. doi: 10.1016/j.energy.2025.138640

[28] Nie B, Liu X, Yang L, Meng J, Li X. Pore structure characterization of different rank coals using gas adsorption and scanning electron microscopy. Fuel. 2015;158:908–17. doi: 10.1016/j.fuel.2015.06.050

[29] He H, Liu P, Xu L, Hao S, Qiu X, Shan C, et al. Pore structure representations based on nitrogen adsorption experiments and an FHH fractal model: Case study of the block Z shales in the Ordos Basin, China. J Pet Sci Eng. 2021;203:108661. doi: 10.1016/j.petrol.2021.108661

[30] Thommes M, Kaneko K, Neimark AV, Olivier JP, Rodriguez-Reinoso F, Rouquerol J. Physisorption of gases, with special reference to the evaluation of surface area and pore size distribution. Pure Appl Chem. 2015;87(9–10):1051–69. doi: 10.1515/pac-2014-1117

[31] Zhu Z, Wu F, Song X, Cheng W, Sanchez-Ferrer A, Zhao J. Characterizing the pore structure of wood materials: A conjoint analysis based on mercury intrusion porosimetry, N2 adsorption and X-ray computed tomography methods. J Clean Prod. 2025;525:146652. doi: 10.1016/j.jclepro.2025.146652

[32] Qin L, Li S, Zhai C, Lin H, Zhao P, Yan M, et al. Joint analysis of pores in low, intermediate, and high rank coals using mercury intrusion, nitrogen adsorption, and nuclear magnetic resonance. Powder Technol. 2020;362:615–27. doi: 10.1016/j.powtec.2019.12.019

[33] Ni G, Li S, Rahman S, Xun M, Wang H, Xu Y, et al. Effect of nitric acid on the pore structure and fractal characteristics of coal based on the low-temperature nitrogen adsorption method. Powder Technol. 2020;367:506–16. doi: 10.1016/j.powtec.2020.04.011

[34] Xu C, Li H, Lu Y, Liu T, Lu J, Shi S, et al. Influence of microwave-assisted oxidant stimulation on pore structure and fractal characteristics of bituminous coal based on low-temperature nitrogen adsorption. Fuel. 2022;327:125173. doi: 10.1016/j.fuel.2022.125173

[35] Rouquerol J, Rouquerol F, Llewellyn P, Maurin G, Sing K. Adsorption by powders and porous solids: Principles, methodology and applications. 2nd ed. Oxford: Academic Press; 2013.

[36] Washburn EW. The dynamics of capillary flow. Phys Rev. 1921;17(3):273–83. doi: 10.1103/PhysRev.17.273

[37] Washburn EW. Note on a method of determining the distribution of pore sizes in a porous material. Proc Natl Acad Sci U S A. 1921;7(4):115–6. doi: 10.1073/pnas.7.4.115 16576588 PMC1084764

[38] León y León CA. New perspectives in mercury porosimetry. Adv Colloid Interface Sci. 1998;76–77:341–72. doi: 10.1016/s0001-8686(98)00052-9

[39] Kaufmann J, Loser R, Leemann A. Analysis of cement-bonded materials by multi-cycle mercury intrusion and nitrogen sorption. J Colloid Interface Sci. 2009;336(2):730–7. doi: 10.1016/j.jcis.2009.05.029 19505695

[40] Liu C, Sang S, Zhang K, Song F, Wang H, Fan X. Effects of temperature and pressure on pore morphology of different rank coals: Implications for CO2 geological storage. J CO2 Util. 2019;34:343–52. doi: 10.1016/j.jcou.2019.07.025

[41] Avnir D, Jaroniec M. An isotherm equation for adsorption on fractal surfaces of heterogeneous porous materials. Langmuir. 1989;5(6):1431–3. doi: 10.1021/la00090a032

[42] Neimark AV. Calculating surface fractal dimensions of adsorbents. Adsorpt Sci Technol. 1990;7(4):210–9. doi: 10.1177/026361749000700402

[43] Zhang X, Cheng J, Zhang L, Zhou T, Kang T, Li L. Pore fractal characteristics of suancigou long-flame coal after electrochemical treatment: an experimental study through the implementation of N2 adsorption and mercury intrusion prosimetry techniques. ACS Omega. 2021;6(41):27358–67. doi: 10.1021/acsomega.1c04231 34693156 PMC8529665

[44] Zhang J, Lu X, Tian B, Chen B, Tan T, Li H, et al. Exploration of macro-micro damage and pore structure fractal of low-calcium fly ash-based concrete (LFC) based on Menger Sponge model in dry-wet and freeze-thaw synergistic environment. J Build Eng. 2025;106:112599. doi: 10.1016/j.jobe.2025.112599

[45] Han W, Zhou G, Gao D, Zhang Z, Wei Z, Wang H, et al. Experimental analysis of the pore structure and fractal characteristics of different metamorphic coal based on mercury intrusion‑nitrogen adsorption porosimetry. Powder Technol. 2020;362:386–98. doi: 10.1016/j.powtec.2019.11.092

[46] Naveen P, Asif M, Ojha K. Integrated fractal description of nanopore structure and its effect on CH4 adsorption on Jharia coals, India. Fuel. 2018;232:190–204. doi: 10.1016/j.fuel.2018.05.124

[47] Zheng C, Li J, Xue S, Jiang B, Liu B. Experimental study on changes in components and pore characteristics of acidified coal treated by organic solvents. Fuel. 2023;353:129215. doi: 10.1016/j.fuel.2023.129215

[48] Abelly EN, Yang F, Ngata MR, Mwakipunda GC, Shanghvi ER. A field study of pore-network systems on the tight shale gas formation through adsorption-desorption technique and mercury intrusion capillary porosimeter: Percolation theory and simulations. Energy. 2024;302:131771. doi: 10.1016/j.energy.2024.131771

