# Supplementary material for: Effect of microwave irradiation cycles on the pore structures and fractal dimension of coals with different ranks
Source: PLoS One. 2026 Jul 29;21(7):e0343007. doi: 10.1371/journal.pone.0343007 (PMC13419212; doi:10.1371/journal.pone.0343007)
Supplement: Attachment — Submitted filename: Response to Reviewers.docx [file pone.0343007.s002.docx]

Dear Dr. Xinyuan Gao and reviewers,

Thank you for your letter and the reviewer′s comments on our manuscript entitled “Effect of microwave irradiation cycles on the pore structures and fractal dimension of coals with different ranks” (Manuscript number: PONE-D-26-04593), and we are grateful that you gave our manuscript an opportunity to revise. Those comments are very helpful for revising and improving our manuscript, as well as providing an important guiding significance to other researchers. We have studied the comments carefully and made corrections which we hope will meet with approval. The main corrections are in the manuscript, and the responses to the reviewer’s comments are as follows (the replies are highlighted in blue).

Replies to the comments from reviewer 1:

Comments:

1. The title of the paper is different coal grades, and three samples were selected for the work, which is also good. However, for each coal grade, there is only one sample, which will affect the correctness of the conclusion.

**Response:** Thank you for your meticulous suggestion. We sincerely thank the reviewer for raising this important point regarding sample representativeness. We are fully aware that using only one sample of each coal grade will limit the universality of the conclusion.

In this study, three coal samples, subbituminous coal, bituminous coal, and anthracite coal, were selected as the representatives of low, medium, and high rank coals with different metamorphic degrees, and each coal sample has stable coal quality characteristics. This study aims to conduct a preliminary systematic comparison of how microwave irradiation cycles affect the pore structures of different coal ranks under controlled laboratory conditions. We agreed that the ideal method should include selecting multiple samples for each coal rank to consider the differences between different samples of the same coal rank. This study has this limitation, and the following explanations have been added in the discussion section to clarify this view (Lines 472-474):

“Only one coal sample of subbituminous coal, bituminous coal, and anthracite coal was selected for analysis, and the differences of different samples of the same coal type under microwave irradiation cycles were not studied, so there are some limitations.”

We believe that, despite this limitation, the trends observed in the three coal samples of different ranks under multiple microwave irradiation cycles still provide valuable insights into the microwave-induced evolution of coal pore structures and can serve as an important reference for future research.

2. Microwave pyrolysis has essential differences from other methods, but both can affect pore characteristics and related parameters. It is recommended to further discuss the mechanism of influence.

**Response:** Thank you for your meticulous suggestion. We are very grateful to the examiner for this key recommendation. In the revised manuscript, we compared the differences between microwave and other traditional methods (Lines 432–442). In order to clearly and intuitively show the relevant mechanism, we have drawn a new schematic diagram of the principle of microwave irradiation modification (Fig 14.) according to the experimental results to better clarify the mechanism of the effect of microwave irradiation cycles on coal pore structures. Revised as follows:

“Compared with the traditional pyrolysis process, microwave irradiation technology provides volumetric and selective heating functions, which can realize the transformation of pore structure faster and more energy-saving. Different from chemical methods (such as acid/alkali treatment), microwave treatment is an anhydrous and reagent free treatment method, which can avoid secondary pollution. In order to clarify the mechanism behind microwave-induced pore structure evolution, the modification principle is shown in Figure 14. As shown in the figure, the microwave irradiation cycle has changed the pore structure and connectivity of coal from two aspects. One is that the internal bound water of coal is transformed into high-pressure steam by microwave; The second is that microwave causes thermal stress in the coal, leading to mineral fracture and pore increase.”

**Fig 14. Schematic diagram of microwave irradiation modification principle.**

We believe that the combination of the new schematic and text can clearly clarify the essential differences between microwave pyrolysis and other modification methods, as well as the mechanism of microwave radiation affecting coal pore characteristics.

3. The increase of pore volume and specific surface area of small pores (10<50 nm). Here, is it a mistake. 10-50 nm?

**Response:** Thank you for your meticulous suggestion. The intended expression was indeed “10–50 nm,” and we apologize for the oversight. This has been corrected in the revised manuscript (Line 41).

4. Regarding pore data, has it been validated with other methods. For example, mercury intrusion test.

**Response:** Thank you for your meticulous suggestion. In this study, low temperature nitrogen gas adsorption (LT-N_2_GA) and mercury intrusion porosimetry (MIP) were both employed to characterize the coal pore structures. These two methods are complementary: LT-N_2_GA is well-suited for micropores (<10 nm) and small pores (10-50 nm), while MIP is more appropriate for macropores (>50 nm). The combined application of both techniques allows for a more comprehensive assessment of the pore size distribution across the full range.

We have also added a brief statement in the Introduction section (Lines 111-116) to clarify the complementary roles of these two methods:

“In order to ensure the comprehensive characterization of pore structure, the low temperature N_2_ adsorption method was used to characterize the micropores and small pores of coal samples, and the mercury intrusion porosimetry method was used to characterize the changes of macropores of coal samples. The combined application of the two methods is helpful to cross verify the evolution of pore structure caused by microwave irradiation cycles.”

We thank the reviewer for encouraging us to highlight this validation.

5. The time and frequency of microwave pyrolysis are very important. It is recommended that the author conduct more experiments for comparison.

**Response:** Thank you for your meticulous suggestion. We fully agree that microwave irradiation time and output power are critical parameters that significantly influence the pore structures modification of coal.

In the current study, the microwave frequency was fixed at 2.45 GHz, with a single irradiation duration of 120 s and an output power of 700 W per cycle (Lines 174-175 in the manuscript). These parameters were selected based on preliminary experiments and established protocols to ensure effective energy input while avoiding excessive carbonization. The primary focus of this investigation was the number of microwave irradiation cycles (1, 3, 5, and 10 cycles), which allowed us to systematically evaluate the cumulative effect of repeated microwave irradiation on pore structures of different coal ranks.

We acknowledge that a more comprehensive experimental design would include systematic variations of irradiation time and power levels to fully optimize microwave treatment conditions. In order to make up for this limitation, we added a statement in the conclusion (lines 475–479), which clearly pointed out that the synergistic effect of irradiation time, power, and cycle number should be explored in subsequent studies, so as to further explore the stimulating effect of microwave on coal pore structures:

“Under the condition of constant irradiation time (120 s) and output power (700 W), the influence of the number of irradiation cycles on the irradiation effect was studied. Microwave radiation time and power are also the key parameters affecting the evolution of coal pore structure. In the future, the synergistic effect of irradiation time, power, and cycle times on coal pore structure can be further discussed by combining the variables of time and power.”

We appreciate the reviewer’s constructive suggestion, which will help guide our future research directions.

Replies to the comments from reviewer 2:

Comments:

1. In the Introduction, the domestic and international research progress on microwave modification of coal has not been systematically reviewed, and the limitations of existing studies are not clearly summarized. The innovation of this study regarding microwave irradiation cycles, different coal ranks, and pore‑fractal combined characterization should be better emphasized. In addition, the statement of research significance is too general; please specify the engineering value of this work by addressing the key challenges in low‑permeability coalbed methane recovery.

**Response:** Thank you for your meticulous suggestion. We sincerely thank the reviewer for this thorough and constructive comment. We agree that the original introduction fails to fully explain the research background, summarize the limitations of existing studies, and clearly clarify the innovation and engineering significance of this study. In response, we have substantially rewritten the Introduction section to address these concerns. The key revisions are summarized below:

(1) Research progress and limitations of microwave modification of coal

We have expanded the literature review to provide a more comprehensive overview of microwave modified coal. The limitations of existing studies are also clearly summarized (Lines 63–80):

“Dong et al. [18] studied the effect of microwave on coal gasification by using synchronous thermal analyzer, Fourier transform infrared spectrometer, and quantum chemical calculation. The results showed that microwave drying could increase the gasification reactivity of coal and improve the performance of underground coal gasification. Qi et al. [19] studied the damage and seepage characteristics of water saturated coal under the action of microwave. The results showed that microwave treatment had a good effect on enhancing permeability, and the initial permeability, failure permeability, and average permeability of coal samples were improved. Huang et al. [20] conducted a comparative study on microwave and conventional pyrolysis of tar-rich coal. They found that microwave irradiation excites oxygen-containing groups and aromatic rings in coal, generating heat that cleaves macromolecules into smaller compounds and promotes radical formation. Additionally, microwave pyrolysis reduces the oxygen content in tar through functional group migration and accelerates aliphatic conversion to radicals for H2/CO. According to Chemerinskiy et al. [21], the use of microwave technology can modify the coal metamorphic stage, which in turn enhances coal processing efficiency, lowers energy consumption, and minimizes hazardous emissions. Most of the existing researches focus on the macro phenomena of microwave treatment on coal, such as the improvement of permeability, gasification efficiency, pyrolysis characteristics, and coal rank transformation. However, the pore structures evolution of coal under microwave irradiation, especially the effect of different microwave cycles on the pore structures of different coal ranks, has not been fully studied.”

(2) Enhanced emphasis on innovation

We have reorganized the description of the innovative method of this study in the Introduction to clearly highlight the innovation of this study in terms of microwave irradiation cycles, different coal ranks, and pore‑fractal combined characterization(Lines 117–126):

“Although the pore structure of coal has been widely studied, the joint characterization of pore structure analysis results and fractal dimension evaluation, especially in different microwave irradiation cycles and between different coal ranks, is rarely reported. Fractal dimension analysis can provide a quantitative expression for the pore surface irregularity and pore size distribution heterogeneity, which is more conducive to understanding the micro level of the effect of microwave cycles on coal pore structure. In order to fill these gaps, the microwave irradiation cycles, pore structure, and fractal dimension of three coal ranks (subbituminous coal, bituminous coal, and anthracite coal) were jointly characterized in this study. In order to comprehensively understand the evolution process of pore structure of different ranks of coal under periodic microwave irradiation, the low-temperature N_2_ adsorption method, mercury invasive porosity measurement method, and fractal theory were used.”

(2) Specific engineering significance of low permeability CBM recovery

We have rewritten the statement of research significance in the Introduction to directly address the key engineering challenges in low permeability coal bed methane (CBM) mining. The revised text is now stated as follows(Lines 127–137):

“Low permeability coal reservoirs pose a major challenge to efficient CBM Recovery because traditional stimulation methods, such as hydraulic fracturing, often face many limitations, including water sensitivity, formation damage, and environmental concerns. Microwave irradiation provides an anhydrous and non-invasive stimulation means, which can induce thermal micro fracture and enhance pore connectivity, so as to improve the permeability of coal and promote CBM desorption. However, the successful application of this technology requires a systematic understanding of how the microwave processing parameters, especially the irradiation period, affect the evolution of pore structure in different coal ranks. The purpose of this study is to meet this demand by clarifying the response of three typical coals to microwave irradiation cycles on the pore scale. This provides a scientific basis for optimizing intermittent microwave stimulation strategy to enhance the gas production capacity of low permeability CBM reservoir.”

We believe that these amendments have greatly improved the Introduction part. By reviewing the research progress of microwave modification of coal, we clearly pointed out the limitations of the current research, highlighted the innovation, and elaborated on the engineering significance. We thank the reviewer for their valuable guidance, which has played an important role in strengthening this key chapter.

2. In the Experimental procedure, microwave irradiation was applied for 1, 3, 5, and 10 cycles, but the justification for selecting these cycle numbers is missing. It is recommended to supplement relevant literature support or preliminary experimental evidence to improve the scientific rigor of the experimental design.

**Response:** Thank you for your meticulous suggestion. We sincerely thank the commentator for his professional opinion. Our decision to select 1, 3, 5, and 10 irradiation cycles is based on two considerations: the support of relevant literature and engineering practicability.

Firstly, the selected number of cycles (1, 3, 5, and 10) is determined based on the range used in the existing research on coal microwave cycle irradiation. For example, Dong et al. [1] when studying the effect of microwave on coal gasification process, set the number of microwave irradiation experiments as 0, 1, 2, 3, 4. Qi et al. [2] when studying the damage and seepage characteristics of water saturated coal under the action of microwave, set the microwave times as 0, 2, 4, 6. Our experimental range (1, 3, 5, and 10 cycles) covers not only the low to medium number of cycles in the above study, but also the higher number of cycles (10 cycles). This expansion enables us to capture the cumulative effect beyond the scope of previous studies, thus providing new insights for long-term or repetitive microwave stimulation. In addition, in the field application of microwave-assisted CBM recovery, intermittent or cyclic treatment may be more practical and energy-saving than continuous irradiation. From single irradiation (1 cycle) to moderate repetition (3 and 5 cycles) and prolonged irradiation (10 cycles), it represents a gradual scenario simulating repeated microwave stimulation in real operation. This range enables us to systematically evaluate the change of pore structure with the increase of cumulative microwave exposure.

In the revised manuscript, we added a reasonable explanation for selecting 1, 3, 5, and 10 irradiation cycles in the experimental part (Lines 172-177):

“This choice is based on the range used in the previous study on Microwave cycle irradiation of coal (for example, 0, 1, 2, 3, 4 in Dong et al. [18] and 0, 2, 4, 6 in Qi et al. [19]), and it also extends the range to a higher number of cycles (10 cycles) to study the impact on coal beyond the previous number of cycles. This range also represents a gradual scheme of intermittent microwave stimulation in practical application, which can systematically evaluate the evolution of pore structure with the increase of cumulative microwave irradiation.”

We believe that this supplement improves the scientific precision and recognition of the experimental design. We thank the reviewers for helping us improve the manuscript of this part.

References

(1) Dong M, Feng L, Qin B. Characteristics of coal gasification with CO2 after microwave irradiation based on TGA, FTIR, and DFT theory. Energy. 2023 Mar 15;267:126619.

(2) Qi X, Liu X, Ma H, Liu Z, Xie W, Zhang Y, et al. Study on the damage and seepage characteristics of water-saturated coal by microwave cycling. Scientific Reports. 2024 Jul 30;14(1):17514.

3. Although various pore size classification methods are introduced in the Introduction, the specific pore size classification criteria used in this study are not described.

**Response:** Thank you for your meticulous suggestion. We thank the reviewer for pointing out this lack of clarity. In the original manuscript, we only mentioned the names of the two pore size classification methods—the former Soviet Union's Hodote classification and the IUPAC classification—without providing their specific definitions, as well as the reasons for the pore size classification in this study, which may cause readers to confuse the pore size standards actually adopted in this study. We apologize for this oversight.

In the revised manuscript, we have expanded the Introduction (lines 97–106) to clearly present the specific pore size classification criteria and reasons adopted in this study as follows:

“In 1961, in order to completely reveal the nature of coal as a gas aggregate, Hodote of the former Soviet Union proposed the classification criteria of pores in coal: micropores (<10 nm), small pores (10-100 nm), mesopores (100-1000 nm), and macropores (>1000 nm) [26]. In 1985, the International Union of Pure and Applied Chemistry (IUPAC) proposed the classification scheme of pores in solid media as micropores (<2 nm), mesopores (2-50 nm), and macropores (>50 nm) [27]. The low temperature N2 adsorption method can test the pores with pore size of about 1.7 nm~300 nm, but it is not accurate enough to test the pores with pore size greater than 50 nm in practical application; The mercury intrusion porosimetry has a wide range of measurement, covering the pores of nanometer and micrometer scale, but mercury intrusion under high pressure is easy to cause damage to the pore structure, which leads to the inaccuracy of mercury porosimetry in the determination of pores with pore size less than 50 nm.”

We appreciate the reviewer’s patience and thank you for helping us improve the completeness and clarity of our manuscript.

4. On lines 264–265, the sentence “the pore volume and specific surface area of micropores decreased, and the pore volume and specific surface area of micropores increased” is erroneous and self‑contradictory.

**Response:** Thank you for your meticulous suggestion. We apologize for this mistake, which was caused by an inadvertent editing oversight. This error has been corrected in the revised manuscript (Lines 313–314). The corrected sentence now reads:

“After microwave irradiation cycles, the pore volume and specific surface area of micropores decreased, and the pore volume and specific surface area of small pores increased.”

We have also carefully reviewed the entire manuscript to ensure no similar errors remain. We appreciate the reviewer’s diligence in helping us improve the accuracy of our manuscript.

5. In the section FHH fractal dimensions of coal samples, only the evolution of fractal dimensions for the same coal under different cycle numbers is analyzed. It is suggested to add cross‑comparison and mechanistic analysis of fractal dimensions among different coal ranks (subbituminous, bituminous, anthracite) at the same irradiation cycle.

**Response:** Thank you for your meticulous suggestion. We strongly agree that the cross-comparison of fractal dimensions of different coal ranks at the same irradiation cycle can provide valuable insights into the role of microwave cycles in the modification of coal pore structure.

In the revised manuscript, we have added a new paragraph in the FHH fractal dimensions section (Lines 333–333) to address this comment. The additions include:

“As shown in the figure, for the raw coal samples, the irregularity of micropores and small pores surfaces follows the order: subbituminous coal sample>bituminous coal sample>anthracite coal sample. After 10 microwave irradiation cycles, D_1_ gradually decreases, indicating a reduction in the surface irregularity of micropores and small pores across all coal samples. The degree of reduction follows the order: bituminous coal sample>subbituminous coal sample>anthracite coal sample, with decreases of 12.13%, 6.02%, and 3.93%, respectively. Regarding the irregularity of pore size distribution in micropores and small pores, the raw coal samples show the following order: anthracite coal sample>bituminous coal sample>subbituminous coal sample. After 10 microwave irradiation cycles, D_2_ slowly decreases, indicating a reduction in the irregularity of pore size distribution for micropores and small pores. The degree of reduction follows the order: anthracite coal sample>bituminous coal sample>subbituminous coal sample, with decreases of 3.01%, 2.76%, and 1.31%, respectively.”

We believe these additions significantly strengthen the fractal analysis by providing both cross‑comparative data and mechanistic interpretation. We thank the reviewer for guiding us to improve the depth of our analysis.

Once again, thank you very much for your constructive comments and suggestions, which would help us in depth to improve the quality of the manuscript.

Kind regards.

Yuefang Wang

E-mail address: [wyftc@imut.edu.cn](mailto:wyftc@imut.edu.cn)

2026.4.8
