# Supplementary material for: Effect of microwave irradiation cycles on the pore structures and fractal dimension of coals with different ranks
Source: PLoS One. 2026 Jul 29;21(7):e0343007. doi: 10.1371/journal.pone.0343007 (PMC13419212; doi:10.1371/journal.pone.0343007)
Supplement: Attachment — Submitted filename: Response_to_Reviewers_auresp_2.docx [file pone.0343007.s003.docx]

Dear Dr. Xinyuan Gao and reviewers,

Thank you for your letter and the reviewer′s comments on our manuscript entitled “Effect of microwave irradiation cycles on the pore structures and fractal dimension of coals with different ranks” (Manuscript number: PONE-D-26-04593), and we are grateful that you gave our manuscript an opportunity to revise. Those comments are very helpful for revising and improving our manuscript, as well as providing an important guiding significance to other researchers. We have studied the comments carefully and made corrections which we hope will meet with approval. The main corrections are in the manuscript, and the responses to the reviewer’s comments are as follows (the replies are highlighted in blue).

Replies to the comments from reviewer 3:

Comments:

1. Although the paper is titled "Effect of microwave irradiation cycles on the pore structures and fractal dimension of coals with different ranks," it does not identify the specific reasons underlying the differences among different coal ranks. It only supplements quantitative data from cross-comparisons of fractal dimensions among different coal ranks, while the mechanistic analysis remains relatively thin. It is therefore recommended to optimize Fig. 14 by adding a schematic illustration of the differential responses among different coal ranks.

**Response:** Thank you for this valuable and constructive suggestion. We agree that the original manuscript did not sufficiently clarify the specific reasons for the differences in pore structure responses among coals of different ranks under microwave irradiation cycles.

To address this issue, a new section entitled “Microwave irradiation mechanism” has been added to the manuscript, and the corresponding content has been substantially expanded. By combining the compositional characteristics of the three types of coal presented in Table 1 with the pore structure evolution and fractal dimension results shown in Figs 5-13, the revised text explains the different responses of subbituminous coal, bituminous coal, and anthracite. This revision clarifies why the same microwave irradiation cycle treatment leads to different degrees of pore expansion, macropore development, and fissures evolution among the three coal ranks.

In addition, Fig 14 has been redesigned by incorporating a schematic illustration of the rank dependent pore and fracture response mechanisms of subbituminous coal, bituminous coal, and anthracite. The revised figure now highlights the distinct response pathways of the three coal ranks. The newly added content is as follows: (Lines 452-471)

“The differences in the responses of coals of different ranks to microwave irradiation cycles can be further explained by combining their basic properties with the evolution of pore structure and fractal characteristics. Subbituminous coal has the highest moisture content (13.47%) and oxygen content (14.12%). After 10 cycles, its maximum mercury intrusion amount increases only from 0.1253 to 0.1501 cm3/g, which is the smallest increase among the three coals, whereas D4 shows the most pronounced increase. This indicates that the response of subbituminous coal is more closely related to pore expansion caused by steam pressure. It is mainly manifested by the transformation of micropores into small pores, accompanied by limited fissures extension, while the overall growth of macropores remains relatively weak. Bituminous coal shows the strongest overall response. Its maximum mercury intrusion amount increases from 0.1233 to 0.3987 cm3/g, and D1 exhibits the largest decrease, suggesting the most evident smoothing of pore surfaces, development of macropores, and enhancement of fissures connectivity. This implies a stronger combined effect of steam pressure expansion and heterogeneous thermal stress. Anthracite has the lowest volatile matter content (6.71%) and oxygen content (3.91%), but a relatively high ash yield (10.75%). Its maximum mercury intrusion amount increases from 0.1662 to 0.3389 cm3/g, and the decrease in D3 is the greatest, while the increase in D4 is relatively limited. These results suggest that anthracite is characterized more by redistribution of pore sizes and thermal stress induced reorganization of macropores and fissures within a dense matrix, in which thermal mismatch between minerals and the coal matrix may play a more important role.

**Fig 14. Schematic diagram of microwave irradiation modification principle.**”

We believe that these revisions strengthen the mechanistic interpretation of the experimental results and make the manuscript more consistent with its title and research objective.

2. The research objective of this paper is to provide a theoretical basis for enhancing low-permeability coalbed methane (CBM) recovery. However, it only analyzes the variation patterns of pore structure and fractal dimensions, without elucidating the mechanisms by which these changes influence CBM transport. It is recommended to incorporate relevant discussion in the Conclusion section to clarify the engineering application value of the findings.

**Response:** Thank you for this valuable suggestion. We agree that the engineering significance of the pore-structure evolution induced by microwave irradiation cycles should be more clearly connected to low-permeability CBM recovery. Accordingly, a new item (5) has been added to the Conclusion section. Based on the pore-structure results obtained in this study, the revised conclusion clarifies that microwave irradiation cycles promote the transformation of micropores into small pores, increase macropore development, and enhance the connectivity of macroporous fissures. These changes may contribute to the formation of more continuous multiscale pore–fracture transport pathways in coal, thereby providing a structural basis for improving CBM migration in low-permeability reservoirs. Revised as follows: (Lines 508-513)

“(5) From the perspective of low-permeability CBM reservoir stimulation, microwave irradiation cycles reconstruct the multiscale pore–fracture system of coal by promoting the transformation of micropores into small pores, increasing the development of macropores, and enhancing the connectivity of macroporous fissures. These structural changes may facilitate the formation of more continuous gas transport pathways within coal and provide a pore-structure basis for improving CBM migration under microwave cyclic modification.”

3. In Conclusion (3), the phrase "with the increase of microwave frequency" is incorrect and should be changed to "with the increase in the number of microwave irradiation cycles."

**Response:** Thank you for your meticulous suggestion. The phrase “with the increase of microwave frequency” has been corrected to “with the increase in the number of microwave irradiation cycles”, and we apologize for the oversight. This has been corrected in the revised manuscript (Line 495).

4. Items (5) and (6) in the Conclusion section address research limitations and future research directions, and they should not be placed there.

**Response:** Thank you for your meticulous suggestion. We agree with the reviewer's comments. Items (5) and (6) in the original conclusion section, which addressed the limitations of this study and future research directions, have been deleted. These contents have been integrated and moved to the last paragraph before the conclusion section (Lines 475-482). Revised as follows:

“It should be noted that this study selected only one representative sample each of subbituminous coal, bituminous coal, and anthracite coal for analysis; therefore, the possible differences among samples of the same coal rank under microwave irradiation cycles were not further examined. In addition, under fixed irradiation conditions of 120 s and 700 W, this work mainly focused on the effect of the number of irradiation cycles on coal pore structure evolution. Since microwave irradiation time and output power are also important factors influencing the modification effect, future studies should further investigate the synergistic effects of irradiation duration, microwave power, and cycle number on the evolution of coal pore structures.”

5. References [15] and [21] are identical, which constitutes a duplicate citation.

**Response:** Thank you for pointing out this issue. References [15] and [21] are duplicate citations, and we apologize for this oversight. The duplicate references have been removed, and the reference list has been revised (Lines 550-552).

6. The text labels in Fig. 2 should maintain consistent capitalization. Please check the entire manuscript for similar issues and revise accordingly.

**Response:** Thank you for the suggestion. The capitalization of the labels in Fig. 2 has been standardized, and the entire manuscript has been checked for similar inconsistencies. The revised Figures have been re-uploaded.

Once again, thank you very much for your constructive comments and suggestions, which would help us in depth to improve the quality of the manuscript.

Kind regards.

Yuefang Wang

E-mail address: [wyftc@imut.edu.cn](mailto:wyftc@imut.edu.cn)

2026.6.4
