# Supplementary material for: Effect of microwave irradiation cycles on the pore structures and fractal dimension of coals with different ranks
Source: PLoS One. 2026 Jul 29;21(7):e0343007. doi: 10.1371/journal.pone.0343007 (PMC13419212; doi:10.1371/journal.pone.0343007)
Supplement: Peer review history

# Peer review: Decision Letter 0

**DOI:** 10.1371/journal.pone.0343007.r001

**Authors:** Xinyuan Gao

23 Mar 2026

PONE-D-26-04593

Effect of microwave irradiation cycles on the pore structures and fractal dimension of coals with different ranks

PLOS One

Dear Dr. Wang,

Thank you for submitting your manuscript to PLOS ONE. After careful consideration, we feel that it has merit but does not fully meet PLOS ONE’s publication criteria as it currently stands. Therefore, we invite you to submit a revised version of the manuscript that addresses the points raised during the review process.

Please submit your revised manuscript by May 07 2026 11:59PM. If you will need more time than this to complete your revisions, please reply to this message or contact the journal office at plosone@plos.org. When you're ready to submit your revision, log on to https://www.editorialmanager.com/pone/ and select the 'Submissions Needing Revision' folder to locate your manuscript file.

Please include the following items when submitting your revised manuscript:

- A letter that responds to each point raised by the academic editor and reviewer(s). You should upload this letter as a separate file labeled 'Response to Reviewers'.
- A marked-up copy of your manuscript that highlights changes made to the original version. You should upload this as a separate file labeled 'Revised Manuscript with Track Changes'.
- An unmarked version of your revised paper without tracked changes. You should upload this as a separate file labeled 'Manuscript'.

If you would like to make changes to your financial disclosure, please include your updated statement in your cover letter. Guidelines for resubmitting your figure files are available below the reviewer comments at the end of this letter.

If applicable, we recommend that you deposit your laboratory protocols in protocols.io to enhance the reproducibility of your results. Protocols.io assigns your protocol its own identifier (DOI) so that it can be cited independently in the future. For instructions see: https://journals.plos.org/plosone/s/submission-guidelines#loc-laboratory-protocols. Additionally, PLOS ONE offers an option for publishing peer-reviewed Lab Protocol articles, which describe protocols hosted on protocols.io. Read more information on sharing protocols at https://plos.org/protocols?utm_medium=editorial-email&utm_source=authorletters&utm_campaign=protocols.

We look forward to receiving your revised manuscript.

Kind regards,

Xinyuan Gao

Academic Editor

PLOS One

Journal Requirements:

When submitting your revision, we need you to address these additional requirements.

1. Please ensure that your manuscript meets PLOS ONE's style requirements, including those for file naming. The PLOS ONE style templates can be found at

https://journals.plos.org/plosone/s/file?id=wjVg/PLOSOne_formatting_sample_main_body.pdf and

https://journals.plos.org/plosone/s/file?id=ba62/PLOSOne_formatting_sample_title_authors_affiliations.pdf

2. We note that the grant information you provided in the ‘Funding Information’ and ‘Financial Disclosure’ sections do not match.

When you resubmit, please ensure that you provide the correct grant numbers for the awards you received for your study in the ‘Funding Information’ section.

3. Thank you for stating the following financial disclosure:

“Funding Statement Project supported by the National Natural Science Foundation (No. 42502160), Natural Science Foundation of Inner Mongolia (No. 2024QN04006; No. 2025LHMS0523), Basic Research Funds for Directly Affiliated Universities in Inner Mongolia (No. JY20250100), and Research Support Projects for Talent Introduction at the Autonomous Region Level in 2022 (No. DC2300001429).”

Please state what role the funders took in the study.  If the funders had no role, please state: "The funders had no role in study design, data collection and analysis, decision to publish, or preparation of the manuscript."

If this statement is not correct you must amend it as needed.

Please include this amended Role of Funder statement in your cover letter; we will change the online submission form on your behalf.

4. We note that your Data Availability Statement is currently as follows: “All relevant data are within the manuscript and its Supporting Information files.”

Please confirm at this time whether or not your submission contains all raw data required to replicate the results of your study. Authors must share the “minimal data set” for their submission. PLOS defines the minimal data set to consist of the data required to replicate all study findings reported in the article, as well as related metadata and methods (https://journals.plos.org/plosone/s/data-availability#loc-minimal-data-set-definition).

For example, authors should submit the following data:

- The values behind the means, standard deviations and other measures reported;

- The values used to build graphs;

- The points extracted from images for analysis.

Authors do not need to submit their entire data set if only a portion of the data was used in the reported study.

If your submission does not contain these data, please either upload them as Supporting Information files or deposit them to a stable, public repository and provide us with the relevant URLs, DOIs, or accession numbers. For a list of recommended repositories, please see https://journals.plos.org/plosone/s/recommended-repositories.

If there are ethical or legal restrictions on sharing a de-identified data set, please explain them in detail (e.g., data contain potentially sensitive information, data are owned by a third-party organization, etc.) and who has imposed them (e.g., an ethics committee). Please also provide contact information for a data access committee, ethics committee, or other institutional body to which data requests may be sent. If data are owned by a third party, please indicate how others may request data access.

5. PLOS requires an ORCID iD for the corresponding author in Editorial Manager on papers submitted after December 6th, 2016. Please ensure that you have an ORCID iD and that it is validated in Editorial Manager. To do this, go to ‘Update my Information’ (in the upper left-hand corner of the main menu), and click on the Fetch/Validate link next to the ORCID field. This will take you to the ORCID site and allow you to create a new iD or authenticate a pre-existing iD in Editorial Manager.

6. We note that Figure 1 in your submission contain map images which may be copyrighted. All PLOS content is published under the Creative Commons Attribution License (CC BY 4.0), which means that the manuscript, images, and Supporting Information files will be freely available online, and any third party is permitted to access, download, copy, distribute, and use these materials in any way, even commercially, with proper attribution. For these reasons, we cannot publish previously copyrighted maps or satellite images created using proprietary data, such as Google software (Google Maps, Street View, and Earth). For more information, see our copyright guidelines: http://journals.plos.org/plosone/s/licenses-and-copyright.

We require you to either (1) present written permission from the copyright holder to publish these figures specifically under the CC BY 4.0 license, or (2) remove the figures from your submission:

1. You may seek permission from the original copyright holder of Figure(s) [#] to publish the content specifically under the CC BY 4.0 license.

We recommend that you contact the original copyright holder with the Content Permission Form (http://journals.plos.org/plosone/s/file?id=7c09/content-permission-form.pdf) and the following text:

“I request permission for the open-access journal PLOS ONE to publish XXX under the Creative Commons Attribution License (CCAL) CC BY 4.0 (http://creativecommons.org/licenses/by/4.0/). Please be aware that this license allows unrestricted use and distribution, even commercially, by third parties. Please reply and provide explicit written permission to publish XXX under a CC BY license and complete the attached form.”

Please upload the completed Content Permission Form or other proof of granted permissions as an "Other" file with your submission.

In the figure caption of the copyrighted figure, please include the following text: “Reprinted from [ref] under a CC BY license, with permission from [name of publisher], original copyright [original copyright year].”

2. If you are unable to obtain permission from the original copyright holder to publish these figures under the CC BY 4.0 license or if the copyright holder’s requirements are incompatible with the CC BY 4.0 license, please either i) remove the figure or ii) supply a replacement figure that complies with the CC BY 4.0 license. Please check copyright information on all replacement figures and update the figure caption with source information. If applicable, please specify in the figure caption text when a figure is similar but not identical to the original image and is therefore for illustrative purposes only.

The following resources for replacing copyrighted map figures may be helpful:

USGS National Map Viewer (public domain): http://viewer.nationalmap.gov/viewer/

The Gateway to Astronaut Photography of Earth (public domain): http://eol.jsc.nasa.gov/sseop/clickmap/

Maps at the CIA (public domain): https://www.cia.gov/library/publications/the-world-factbook/index.html and https://www.cia.gov/library/publications/cia-maps-publications/index.html

NASA Earth Observatory (public domain): http://earthobservatory.nasa.gov/

Landsat: http://landsat.visibleearth.nasa.gov/

USGS EROS (Earth Resources Observatory and Science (EROS) Center) (public domain): http://eros.usgs.gov/#

Natural Earth (public domain): http://www.naturalearthdata.com/

7. We note that Figure 2 in your submission contain copyrighted images. All PLOS content is published under the Creative Commons Attribution License (CC BY 4.0), which means that the manuscript, images, and Supporting Information files will be freely available online, and any third party is permitted to access, download, copy, distribute, and use these materials in any way, even commercially, with proper attribution. For more information, see our copyright guidelines: http://journals.plos.org/plosone/s/licenses-and-copyright.

We require you to either (1) present written permission from the copyright holder to publish these figures specifically under the CC BY 4.0 license, or (2) remove the figures from your submission:

1. You may seek permission from the original copyright holder of Figure(s) [#] to publish the content specifically under the CC BY 4.0 license.

We recommend that you contact the original copyright holder with the Content Permission Form (http://journals.plos.org/plosone/s/file?id=7c09/content-permission-form.pdf) and the following text:

“I request permission for the open-access journal PLOS ONE to publish XXX under the Creative Commons Attribution License (CCAL) CC BY 4.0 (http://creativecommons.org/licenses/by/4.0/). Please be aware that this license allows unrestricted use and distribution, even commercially, by third parties. Please reply and provide explicit written permission to publish XXX under a CC BY license and complete the attached form.”

Please upload the completed Content Permission Form or other proof of granted permissions as an "Other" file with your submission.

In the figure caption of the copyrighted figure, please include the following text: “Reprinted from [ref] under a CC BY license, with permission from [name of publisher], original copyright [original copyright year].”

2. If you are unable to obtain permission from the original copyright holder to publish these figures under the CC BY 4.0 license or if the copyright holder’s requirements are incompatible with the CC BY 4.0 license, please either i) remove the figure or ii) supply a replacement figure that complies with the CC BY 4.0 license. Please check copyright information on all replacement figures and update the figure caption with source information. If applicable, please specify in the figure caption text when a figure is similar but not identical to the original image and is therefore for illustrative purposes only.

8. If the reviewer comments include a recommendation to cite specific previously published works, please review and evaluate these publications to determine whether they are relevant and should be cited. There is no requirement to cite these works unless the editor has indicated otherwise.

[Note: HTML markup is below. Please do not edit.]

Reviewers' comments:

Reviewer's Responses to Questions

**Comments to the Author**

1. Is the manuscript technically sound, and do the data support the conclusions?

The manuscript must describe a technically sound piece of scientific research with data that supports the conclusions. Experiments must have been conducted rigorously, with appropriate controls, replication, and sample sizes. The conclusions must be drawn appropriately based on the data presented.

Reviewer #1: Yes

Reviewer #2: Yes

**********

2. Has the statistical analysis been performed appropriately and rigorously?

Reviewer #1: Yes

Reviewer #2: Yes

**********

3. Have the authors made all data underlying the findings in their manuscript fully available?

The PLOS Data policy requires authors to make all data underlying the findings described in their manuscript fully available without restriction, with rare exception (please refer to the Data Availability Statement in the manuscript PDF file). The data should be provided as part of the manuscript or its supporting information, or deposited to a public repository. For example, in addition to summary statistics, the data points behind means, medians and variance measures should be available. If there are restrictions on publicly sharing data—e.g. participant privacy or use of data from a third party—those must be specified.

Reviewer #1: Yes

Reviewer #2: Yes

**********

4. Is the manuscript presented in an intelligible fashion and written in standard English?

PLOS ONE does not copyedit accepted manuscripts, so the language in submitted articles must be clear, correct, and unambiguous. Any typographical or grammatical errors should be corrected at revision, so please note any specific errors here.

Reviewer #1: Yes

Reviewer #2: Yes

**********

5. Review Comments to the Author

Please use the space provided to explain your answers to the questions above. You may also include additional comments for the author, including concerns about dual publication, research ethics, or publication ethics. (Please upload your review as an attachment if it exceeds 20,000 characters)

Reviewer #1: This manuscript is well written, choosing a new method to improve the porosity and obtaining a large amount of pore data. It can be accepted after minor revision.

1 The title of the paper is different coal grades, and three samples were selected for the work, which is also good. However, for each coal grade, there is only one sample, which will affect the correctness of the conclusion.

2 Microwave pyrolysis has essential differences from other methods, but both can affect pore characteristics and related parameters. It is recommended to further discuss the mechanism of influence.

3 The increase of pore volume and specific surface area of small pores (10<50 nm). Here, is it a mistake. 10-50 nm?

4 Regarding pore data, has it been validated with other methods. For example, mercury intrusion test.

5 The time and frequency of microwave pyrolysis are very important. It is recommended that the author conduct more experiments for comparison.

Reviewer #2: This paper experimentally investigates the effect of microwave irradiation cycles on the pore structure and fractal dimension of coals with different ranks. The topic is well aligned with engineering demands for coalbed methane reservoir stimulation and efficient extraction. The research logic is clear, the characterization methods including low‑temperature nitrogen adsorption, mercury intrusion porosimetry, and fractal theory are reasonably employed, the experimental data are relatively complete, and the conclusions offer certain theoretical reference and engineering guidance.

Overall, I support the publication of this work subject to the following comments to be carefully addressed.

1. In the Introduction, the domestic and international research progress on microwave modification of coal has not been systematically reviewed, and the limitations of existing studies are not clearly summarized. The innovation of this study regarding microwave irradiation cycles, different coal ranks, and pore‑fractal combined characterization should be better emphasized. In addition, the statement of research significance is too general; please specify the engineering value of this work by addressing the key challenges in low‑permeability coalbed methane recovery.

2. In the Experimental procedure, microwave irradiation was applied for 1, 3, 5, and 10 cycles, but the justification for selecting these cycle numbers is missing. It is recommended to supplement relevant literature support or preliminary experimental evidence to improve the scientific rigor of the experimental design.

3. Although various pore size classification methods are introduced in the Introduction, the specific pore size classification criteria used in this study are not described.

4. On lines 264–265, the sentence “the pore volume and specific surface area of micropores decreased, and the pore volume and specific surface area of micropores increased” is erroneous and self‑contradictory.

5. In the section FHH fractal dimensions of coal samples, only the evolution of fractal dimensions for the same coal under different cycle numbers is analyzed. It is suggested to add cross‑comparison and mechanistic analysis of fractal dimensions among different coal ranks (subbituminous, bituminous, anthracite) at the same irradiation cycle.

**********

6. PLOS authors have the option to publish the peer review history of their article (what does this mean?). If published, this will include your full peer review and any attached files.

If you choose “no”, your identity will remain anonymous but your review may still be made public.

**Do you want your identity to be public for this peer review?**  For information about this choice, including consent withdrawal, please see our Privacy Policy.

Reviewer #1: No

Reviewer #2: No

**********

[NOTE: If reviewer comments were submitted as an attachment file, they will be attached to this email and accessible via the submission site. Please log into your account, locate the manuscript record, and check for the action link "View Attachments". If this link does not appear, there are no attachment files.]

To ensure your figures meet our technical requirements, please review our figure guidelines: https://journals.plos.org/plosone/s/figures

You may also use PLOS’s free figure tool, NAAS, to help you prepare publication quality figures: https://journals.plos.org/plosone/s/figures#loc-tools-for-figure-preparation.

NAAS will assess whether your figures meet our technical requirements by comparing each figure against our figure specifications.

# Author comment: Author response to Decision Letter 1

**DOI:** 10.1371/journal.pone.0343007.r002

10 Apr 2026

Dear Dr. Xinyuan Gao and reviewers,

Thank you for your letter and the reviewer′s comments on our manuscript entitled “Effect of microwave irradiation cycles on the pore structures and fractal dimension of coals with different ranks” (Manuscript number: PONE-D-26-04593), and we are grateful that you gave our manuscript an opportunity to revise. Those comments are very helpful for revising and improving our manuscript, as well as providing an important guiding significance to other researchers. We have studied the comments carefully and made corrections which we hope will meet with approval.

Reply to editorial comments:

1. Style and Formatting Requirements

Response: Thank you for reminding. We are sorry for not complying with the journal requirements before. We have reviewed the PLOS ONE style requirements and updated the manuscript accordingly. This includes adjustments to the file naming conventions, title page layout, and the main body formatting (e.g., heading styles, font, and spacing) to match the provided templates.

2. Funding Information Consistency

Response: Thank you for pointing this out. We are sorry for not complying with the journal requirements before. Thank you for reminding. We have corrected the ‘Funding Information’ section in the submission system to ensure it matches the grant numbers listed in the manuscript’s ‘Financial Disclosure’.

3. Financial Disclosure and Role of Funders

Response: Thank you for requesting clarification on the role of the funders in our study. The roles of each funder are as follows:

The funder of the National Natural Science Foundation of China (No. 42502160) participated in the study design.

The funding from the Natural Science Foundation of Inner Mongolia (No. 2024QN04006; 2025LHMS0523) supported the sample collection.

The funder of the Basic Research Funds for Directly Affiliated Universities in Inner Mongolia (No. JY20250100) participated in the manuscript writing.

The funder of the Research Support Projects for Talent Introduction at the Autonomous Region Level in 2022(No. DC2300001429) participated in data collection and analysis.

We confirm that this statement accurately reflects the contribution of each funding body.

4. Data Availability Statement

Response: Thank you for reminding. We are sorry for not complying with the journal requirements before. We confirm that our submission now contains all the original data of our research results. We have compiled the values used to build the graphics into Excel files and compressed them (zip), and there is also a table (docx), which has been uploaded as support information files. If you need any further modifications or additional data, please let us know.

5. ORCID iD for Corresponding Author

Response: Thank you for reminding. The ORCID iD for the corresponding author has been validated in the Editorial Manager system via the ‘Update my Information’ link. My ORCID:0009-0003-3872-280X

6. Copyright for Figure 1 (Map Figure)

Response: Thank you for pointing this out. We are sorry for not complying with the journal requirements before. We have replaced the original Fig 1. (which contained proprietary map data) with a new map created using data from Natural Earth (public domain). As Natural Earth data is in the public domain, the new figure fully complies with the PLOS ONE Creative Commons Attribution License (CC BY 4.0). We have updated the caption for Figure 1 to reflect the data source. The revised caption now reads: “Fig 1. Collection of coal samples. The map was generated using public domain data from Natural Earth (http://www.naturalearthdata.com/).”

7. Copyright for Figure 2 (Copyrighted Figure)

Response: Thank you for pointing this out. We are sorry for not complying with the journal requirements before. We have removed the original photograph of the equipment and replaced it with a newly drawn schematic diagram of the device in Fig 2. All elements in the newly drawn diagram are original. The updated figure now fully complies with the copyright policy requirements of PLOS ONE.

We believe these revisions have significantly improved the manuscript and addressed all the concerns raised. We have uploaded the following files:

Cover letter

Response to Reviewers

Revised Manuscript with Track Changes

Manuscript

Supporting Information files: S1 Data. Minimal data set. and S1 Table. Petrologic Characteristics, Elemental Composition, and Proximate Analysis of Coal Samples.

The main corrections are in the manuscript, and the responses to the reviewer’s comments are as follows.

Replies to the comments from reviewer 1:

Comments:

1.The title of the paper is different coal grades, and three samples were selected for the work, which is also good. However, for each coal grade, there is only one sample, which will affect the correctness of the conclusion.

Response: Thank you for your meticulous suggestion. We sincerely thank the reviewer for raising this important point regarding sample representativeness. We are fully aware that using only one sample of each coal grade will limit the universality of the conclusion.

In this study, three coal samples, subbituminous coal, bituminous coal, and anthracite coal, were selected as the representatives of low, medium, and high rank coals with different metamorphic degrees, and each coal sample has stable coal quality characteristics. This study aims to conduct a preliminary systematic comparison of how microwave irradiation cycles affect the pore structures of different coal ranks under controlled laboratory conditions. We agreed that the ideal method should include selecting multiple samples for each coal rank to consider the differences between different samples of the same coal rank. This study has this limitation, and the following explanations have been added in the discussion section to clarify this view (Lines 472-474):

“Only one coal sample of subbituminous coal, bituminous coal, and anthracite coal was selected for analysis, and the differences of different samples of the same coal type under microwave irradiation cycles were not studied, so there are some limitations.”

We believe that, despite this limitation, the trends observed in the three coal samples of different ranks under multiple microwave irradiation cycles still provide valuable insights into the microwave-induced evolution of coal pore structures and can serve as an important reference for future research.

2.Microwave pyrolysis has essential differences from other methods, but both can affect pore characteristics and related parameters. It is recommended to further discuss the mechanism of influence.

Response: Thank you for your meticulous suggestion. We are very grateful to the examiner for this key recommendation. In the revised manuscript, we compared the differences between microwave and other traditional methods (Lines 432–442). In order to clearly and intuitively show the relevant mechanism, we have drawn a new schematic diagram of the principle of microwave irradiation modification (Fig 14.) according to the experimental results to better clarify the mechanism of the effect of microwave irradiation cycles on coal pore structures. Revised as follows:

“Compared with the traditional pyrolysis process, microwave irradiation technology provides volumetric and selective heating functions, which can realize the transformation of pore structure faster and more energy-saving. Different from chemical methods (such as acid/alkali treatment), microwave treatment is an anhydrous and reagent free treatment method, which can avoid secondary pollution. In order to clarify the mechanism behind microwave-induced pore structure evolution, the modification principle is shown in Figure 14. As shown in the figure, the microwave irradiation cycle has changed the pore structure and connectivity of coal from two aspects. One is that the internal bound water of coal is transformed into high-pressure steam by microwave; The second is that microwave causes thermal stress in the coal, leading to mineral fracture and pore increase.”

Fig 14. Schematic diagram of microwave irradiation modification principle.

We believe that the combination of the new schematic and text can clearly clarify the essential differences between microwave pyrolysis and other modification methods, as well as the mechanism of microwave radiation affecting coal pore characteristics.

3.The increase of pore volume and specific surface area of small pores (10<50 nm). Here, is it a mistake. 10-50 nm?

Response: Thank you for your meticulous suggestion. The intended expression was indeed “10–50 nm,” and we apologize for the oversight. This has been corrected in the revised manuscript (Line 41).

4.Regarding pore data, has it been validated with other methods. For example, mercury intrusion test.

Response: Thank you for your meticulous suggestion. In this study, low temperature nitrogen gas adsorption (LT-N2GA) and mercury intrusion porosimetry (MIP) were both employed to characterize the coal pore structures. These two methods are complementary: LT-N2GA is well-suited for micropores (<10 nm) and small pores (10-50 nm), while MIP is more appropriate for macropores (>50 nm). The combined application of both techniques allows for a more comprehensive assessment of the pore size distribution across the full range.

We have also added a brief statement in the Introduction section (Lines 111-116) to clarify the complementary roles of these two methods:

“In order to ensure the comprehensive characterization of pore structure, the low temperature N2 adsorption method was used to characterize the micropores and small pores of coal samples, and the mercury intrusion porosimetry method was used to characterize the changes of macropores of coal samples. The combined application of the two methods is helpful to cross verify the evolution of pore structure caused by microwave irradiation cycles.”

We thank the reviewer for encouraging us to highlight this validation.

5.The time and frequency of microwave pyrolysis are very important. It is recommended that the author conduct more experiments for comparison.

Response: Thank you for your meticulous suggestion. We fully agree that microwave irradiation time and output power are critical parameters that significantly influence the pore structures modification of coal.

In the current study, the microwave frequency was fixed at 2.45 GHz, with a single irradiation duration of 120 s and an output power of 700 W per cycle (Lines 174-175 in the manuscript). These parameters were selected based on preliminary experiments and established protocols to ensure effective energy input while avoiding excessive carbonization. The primary focus of this investigation was the number of microwave irradiation cycles (1, 3, 5, and 10 cycles), which allowed us to systematically evaluate the cumulative effect of repeated microwave irradiation on pore structures of different coal ranks.

We acknowledge that a more comprehensive experimental design would include systematic variations of irradiation time and power levels to fully optimize microwave treatment conditions. In order to make up for this limitation, we added a statement in the conclusion (lines 475–479), which clearly pointed out that the synergistic effect of irradiation time, power, and cycle number should be explored in subsequent studies, so as to further explore the stimulating effect of microwave on coal pore structures:

“Under the condition of constant irradiation time (120 s) and output power (700 W), the influence of the number of irradiation cycles on the irradiation effect was studied. Microwave radiation time and power are also the key parameters affecting the evolution of coal pore structure. In the future, the synergistic effect of irradiation time, power, and cycle times on coal pore structure can be further discussed by combining the variables of time and power.”

We appreciate the reviewer’s constructive suggestion, which will help guide our future research directions.

Replies to the comments from reviewer 2:

Comments:

1.In the Introduction, the domestic and international research progress on microwave modification of coal has not been systematically reviewed, and the limitations of existing studies are not clearly summarized. The innovation of this study regarding microwave irradiation cycles, different coal ranks, and pore‑fractal combined characterization should be better emphasized. In addition, the statement of research significance is too general; please specify the engineering value of this work by addressing the key challenges in low‑permeability coalbed methane recovery.

Response: Thank you for your meticulous suggestion. We sincerely thank the reviewer for this thorough and constructive comment. We agree that the original introduction fails to fully explain the research background, summarize the limitations of existing studies, and clearly clarify the innovation and engineering significance of this study. In response, we have substantially rewritten the Introduction section to address these concerns. The key revisions are summarized below:

(1) Research progress and limitations of microwave modification of coal

We have expanded the literature review to provide a more comprehensive overview of microwave modified coal. The limitations of existing studies are also clearly summarized (Lines 63–80):

“Dong et al. [18] studied the effect of microwave on coal gasification by using synchronous thermal analyzer, Fourier transform infrared spectrometer, and quantum chemical calculation. The results showed that microwave drying could increase the gasification reactivity of coal and improve the performance of underground coal gasification. Qi et al. [19] studied the damage and seepage characteristics of water saturated coal under the action of microwave. The results showed that microwave treatment had a good effect on enhancing permeability, and the initial permeability, failure permeability, and average permeability of coal samples were improved. Huang et al. [20] conducted a comparative study on microwave and conventional pyrolysis of tar-rich coal. They found that microwave irradiation excites oxygen-containing groups and aromatic rings in coal, generating heat that cleaves macromolecules into smaller compounds and promotes radical formation. Additionally, microwave pyrolysis reduces the oxygen content in tar through functional group migration and accelerates aliphatic conversion to radicals for H2/CO. According to Chemerinskiy et al. [21], the use of microwave technology can modify the coal metamorphic stage, which in turn enhances coal processing efficiency, lowers energy consumption, and minimizes hazardous emissions. Most of the existing researches focus on the macro phenomena of microwave treatment on coal, such as the improvement of permeability, gasification efficiency, pyrolysis characteristics, and coal rank transformation. However, the pore structures evolution of coal under microwave irradiation, especially the effect of different microwave cycles on the pore structures of different coal ranks, has not been fully studied.”

(2) Enhanced emphasis on innovation

We have reorganized the description of the innovative method of this study in the Introduction to clearly highlight the innovation of this study in terms of microwave irradiation cycles, different coal ranks, and pore‑fractal combined characterization(Lines 117–126):

“Although the pore structure of coal has been widely studied, the joint characterization of pore structure analysis results and fractal dimension evaluation, especially in different microwave irradiation cycles and between different coal ranks, is rarely reported. Fractal dimension analysis can provide a quantitative expression for the pore surface irregularity and pore size distribution heterogeneity, which is more conducive to understanding the micro level of the effect of microwave cycles on coal pore structure. In order to fill these gaps, the microwave irradiation cycles, pore structure, and fractal dimension of three coal ranks (subbituminous coal, bituminous coal, and anthracite coal) were jointly characterized in this study. In order to comprehensively understand the evolution process of pore structure of different ranks of coal under periodic microwa

Attachment Submitted filename: Response to Reviewers.docx

# Peer review: Decision Letter 1

**DOI:** 10.1371/journal.pone.0343007.r003

**Authors:** Xinyuan Gao

20 May 2026

PONE-D-26-04593R1

Effect of microwave irradiation cycles on the pore structures and fractal dimension of coals with different ranks

PLOS One

Dear Dr. Wang,

Thank you for submitting your manuscript to PLOS ONE. After careful consideration, we feel that it has merit but does not fully meet PLOS ONE’s publication criteria as it currently stands. Therefore, we invite you to submit a revised version of the manuscript that addresses the points raised during the review process.

Please submit your revised manuscript by Jul 04 2026 11:59PM. If you will need more time than this to complete your revisions, please reply to this message or contact the journal office at plosone@plos.org. When you're ready to submit your revision, log on to https://www.editorialmanager.com/pone/ and select the 'Submissions Needing Revision' folder to locate your manuscript file.

Please include the following items when submitting your revised manuscript:

- A letter that responds to each point raised by the academic editor and reviewer(s). You should upload this letter as a separate file labeled 'Response to Reviewers'.
- A marked-up copy of your manuscript that highlights changes made to the original version. You should upload this as a separate file labeled 'Revised Manuscript with Track Changes'.
- An unmarked version of your revised paper without tracked changes. You should upload this as a separate file labeled 'Manuscript'.

If you would like to make changes to your financial disclosure, please include your updated statement in your cover letter. Guidelines for resubmitting your figure files are available below the reviewer comments at the end of this letter.

If applicable, we recommend that you deposit your laboratory protocols in protocols.io to enhance the reproducibility of your results. Protocols.io assigns your protocol its own identifier (DOI) so that it can be cited independently in the future. For instructions see: https://journals.plos.org/plosone/s/submission-guidelines#loc-laboratory-protocols. Additionally, PLOS ONE offers an option for publishing peer-reviewed Lab Protocol articles, which describe protocols hosted on protocols.io. Read more information on sharing protocols at https://plos.org/protocols?utm_medium=editorial-email&utm_source=authorletters&utm_campaign=protocols.

As the corresponding author, your ORCID iD is verified in the submission system and will appear in the published article. PLOS supports the use of ORCID, and we encourage all coauthors to register for an ORCID iD and use it as well. Please encourage your coauthors to verify their ORCID iD within the submission system before final acceptance, as unverified ORCID iDs will not appear in the published article. *Only*  the individual author can complete the verification step; PLOS staff *cannot*  verify ORCID iDs on behalf of authors.

We look forward to receiving your revised manuscript.

Kind regards,

Xinyuan Gao

Academic Editor

PLOS One

Journal Requirements:

If the reviewer comments include a recommendation to cite specific previously published works, please review and evaluate these publications to determine whether they are relevant and should be cited. There is no requirement to cite these works unless the editor has indicated otherwise.

Please review your reference list to ensure that it is complete and correct. If you have cited papers that have been retracted, please include the rationale for doing so in the manuscript text, or remove these references and replace them with relevant current references. Any changes to the reference list should be mentioned in the rebuttal letter that accompanies your revised manuscript. If you need to cite a retracted article, indicate the article’s retracted status in the References list and also include a citation and full reference for the retraction notice.

[Note: HTML markup is below. Please do not edit.]

Reviewers' comments:

Reviewer's Responses to Questions

**Comments to the Author**

1. If the authors have adequately addressed your comments raised in a previous round of review and you feel that this manuscript is now acceptable for publication, you may indicate that here to bypass the “Comments to the Author” section, enter your conflict of interest statement in the “Confidential to Editor” section, and submit your "Accept" recommendation.

Reviewer #1: All comments have been addressed

Reviewer #3: All comments have been addressed

**********

2. Is the manuscript technically sound, and do the data support the conclusions?

The manuscript must describe a technically sound piece of scientific research with data that supports the conclusions. Experiments must have been conducted rigorously, with appropriate controls, replication, and sample sizes. The conclusions must be drawn appropriately based on the data presented.

Reviewer #1: Yes

Reviewer #3: Yes

**********

3. Has the statistical analysis been performed appropriately and rigorously?

Reviewer #1: Yes

Reviewer #3: Yes

**********

4. Have the authors made all data underlying the findings in their manuscript fully available?

The PLOS Data policy requires authors to make all data underlying the findings described in their manuscript fully available without restriction, with rare exception (please refer to the Data Availability Statement in the manuscript PDF file). The data should be provided as part of the manuscript or its supporting information, or deposited to a public repository. For example, in addition to summary statistics, the data points behind means, medians and variance measures should be available. If there are restrictions on publicly sharing data—e.g. participant privacy or use of data from a third party—those must be specified.

Reviewer #1: Yes

Reviewer #3: Yes

**********

5. Is the manuscript presented in an intelligible fashion and written in standard English?

PLOS ONE does not copyedit accepted manuscripts, so the language in submitted articles must be clear, correct, and unambiguous. Any typographical or grammatical errors should be corrected at revision, so please note any specific errors here.

Reviewer #1: Yes

Reviewer #3: Yes

**********

6. Review Comments to the Author

Please use the space provided to explain your answers to the questions above. You may also include additional comments for the author, including concerns about dual publication, research ethics, or publication ethics. (Please upload your review as an attachment if it exceeds 20,000 characters)

Reviewer #1: It has been revised. The author has made the requested revisions, and the manuscript is recommended for acceptance.

Reviewer #3: The authors have undertaken a thorough and conscientious revision, and the manuscript has been substantially improved in response to the previous round of comments. Nevertheless, several issues still need to be addressed before the manuscript can be finalized�

1. Although the paper is titled "Effect of microwave irradiation cycles on the pore structures and fractal dimension of coals with different ranks," it does not identify the specific reasons underlying the differences among different coal ranks. It only supplements quantitative data from cross-comparisons of fractal dimensions among different coal ranks, while the mechanistic analysis remains relatively thin. It is therefore recommended to optimize Fig. 14 by adding a schematic illustration of the differential responses among different coal ranks.

2. The research objective of this paper is to provide a theoretical basis for enhancing low-permeability coalbed methane (CBM) recovery. However, it only analyzes the variation patterns of pore structure and fractal dimensions, without elucidating the mechanisms by which these changes influence CBM transport. It is recommended to incorporate relevant discussion in the Conclusion section to clarify the engineering application value of the findings.

3. In Conclusion (3), the phrase "with the increase of microwave frequency" is incorrect and should be changed to "with the increase in the number of microwave irradiation cycles."

4. Items (5) and (6) in the Conclusion section address research limitations and future research directions, and they should not be placed there.

5. References [15] and [21] are identical, which constitutes a duplicate citation.

6. The text labels in Fig. 2 should maintain consistent capitalization. Please check the entire manuscript for similar issues and revise accordingly.

Overall Recommendation: Minor Revision

**********

7. PLOS authors have the option to publish the peer review history of their article (what does this mean?). If published, this will include your full peer review and any attached files.

If you choose “no”, your identity will remain anonymous but your review may still be made public.

**Do you want your identity to be public for this peer review?**  For information about this choice, including consent withdrawal, please see our Privacy Policy.

Reviewer #1: No

Reviewer #3: **Yes:**  Lei Qin

**********

[NOTE: If reviewer comments were submitted as an attachment file, they will be attached to this email and accessible via the submission site. Please log into your account, locate the manuscript record, and check for the action link "View Attachments". If this link does not appear, there are no attachment files.]

To ensure your figures meet our technical requirements, please review our figure guidelines: https://journals.plos.org/plosone/s/figures

You may also use PLOS’s free figure tool, NAAS, to help you prepare publication quality figures: https://journals.plos.org/plosone/s/figures#loc-tools-for-figure-preparation.

NAAS will assess whether your figures meet our technical requirements by comparing each figure against our figure specifications.

# Author comment: Author response to Decision Letter 2

**DOI:** 10.1371/journal.pone.0343007.r004

3 Jun 2026

Dear Dr. Xinyuan Gao and reviewers,

Thank you for your letter and the reviewer′s comments on our manuscript entitled “Effect of microwave irradiation cycles on the pore structures and fractal dimension of coals with different ranks” (Manuscript number: PONE-D-26-04593), and we are grateful that you gave our manuscript an opportunity to revise. Those comments are very helpful for revising and improving our manuscript, as well as providing an important guiding significance to other researchers. We have studied the comments carefully and made corrections which we hope will meet with approval. The main corrections are in the manuscript, and the responses to the reviewer’s comments are as follows (the replies are highlighted in blue).

Replies to the comments from reviewer 3:

Comments:

1. Although the paper is titled "Effect of microwave irradiation cycles on the pore structures and fractal dimension of coals with different ranks," it does not identify the specific reasons underlying the differences among different coal ranks. It only supplements quantitative data from cross-comparisons of fractal dimensions among different coal ranks, while the mechanistic analysis remains relatively thin. It is therefore recommended to optimize Fig. 14 by adding a schematic illustration of the differential responses among different coal ranks.

Response: Thank you for this valuable and constructive suggestion. We agree that the original manuscript did not sufficiently clarify the specific reasons for the differences in pore structure responses among coals of different ranks under microwave irradiation cycles.

To address this issue, a new section entitled “Microwave irradiation mechanism” has been added to the manuscript, and the corresponding content has been substantially expanded. By combining the compositional characteristics of the three types of coal presented in Table 1 with the pore structure evolution and fractal dimension results shown in Figs 5-13, the revised text explains the different responses of subbituminous coal, bituminous coal, and anthracite. This revision clarifies why the same microwave irradiation cycle treatment leads to different degrees of pore expansion, macropore development, and fissures evolution among the three coal ranks.

In addition, Fig 14 has been redesigned by incorporating a schematic illustration of the rank dependent pore and fracture response mechanisms of subbituminous coal, bituminous coal, and anthracite. The revised figure now highlights the distinct response pathways of the three coal ranks. The newly added content is as follows: (Lines 452-471)

“The differences in the responses of coals of different ranks to microwave irradiation cycles can be further explained by combining their basic properties with the evolution of pore structure and fractal characteristics. Subbituminous coal has the highest moisture content (13.47%) and oxygen content (14.12%). After 10 cycles, its maximum mercury intrusion amount increases only from 0.1253 to 0.1501 cm3/g, which is the smallest increase among the three coals, whereas D4 shows the most pronounced increase. This indicates that the response of subbituminous coal is more closely related to pore expansion caused by steam pressure. It is mainly manifested by the transformation of micropores into small pores, accompanied by limited fissures extension, while the overall growth of macropores remains relatively weak. Bituminous coal shows the strongest overall response. Its maximum mercury intrusion amount increases from 0.1233 to 0.3987 cm3/g, and D1 exhibits the largest decrease, suggesting the most evident smoothing of pore surfaces, development of macropores, and enhancement of fissures connectivity. This implies a stronger combined effect of steam pressure expansion and heterogeneous thermal stress. Anthracite has the lowest volatile matter content (6.71%) and oxygen content (3.91%), but a relatively high ash yield (10.75%). Its maximum mercury intrusion amount increases from 0.1662 to 0.3389 cm3/g, and the decrease in D3 is the greatest, while the increase in D4 is relatively limited. These results suggest that anthracite is characterized more by redistribution of pore sizes and thermal stress induced reorganization of macropores and fissures within a dense matrix, in which thermal mismatch between minerals and the coal matrix may play a more important role.

Fig 14. Schematic diagram of microwave irradiation modification principle.”

We believe that these revisions strengthen the mechanistic interpretation of the experimental results and make the manuscript more consistent with its title and research objective.

2. The research objective of this paper is to provide a theoretical basis for enhancing low-permeability coalbed methane (CBM) recovery. However, it only analyzes the variation patterns of pore structure and fractal dimensions, without elucidating the mechanisms by which these changes influence CBM transport. It is recommended to incorporate relevant discussion in the Conclusion section to clarify the engineering application value of the findings.

Response: Thank you for this valuable suggestion. We agree that the engineering significance of the pore-structure evolution induced by microwave irradiation cycles should be more clearly connected to low-permeability CBM recovery. Accordingly, a new item (5) has been added to the Conclusion section. Based on the pore-structure results obtained in this study, the revised conclusion clarifies that microwave irradiation cycles promote the transformation of micropores into small pores, increase macropore development, and enhance the connectivity of macroporous fissures. These changes may contribute to the formation of more continuous multiscale pore–fracture transport pathways in coal, thereby providing a structural basis for improving CBM migration in low-permeability reservoirs. Revised as follows: (Lines 508-513)

“(5) From the perspective of low-permeability CBM reservoir stimulation, microwave irradiation cycles reconstruct the multiscale pore–fracture system of coal by promoting the transformation of micropores into small pores, increasing the development of macropores, and enhancing the connectivity of macroporous fissures. These structural changes may facilitate the formation of more continuous gas transport pathways within coal and provide a pore-structure basis for improving CBM migration under microwave cyclic modification.”

3. In Conclusion (3), the phrase "with the increase of microwave frequency" is incorrect and should be changed to "with the increase in the number of microwave irradiation cycles."

Response: Thank you for your meticulous suggestion. The phrase “with the increase of microwave frequency” has been corrected to “with the increase in the number of microwave irradiation cycles”, and we apologize for the oversight. This has been corrected in the revised manuscript (Line 495).

4. Items (5) and (6) in the Conclusion section address research limitations and future research directions, and they should not be placed there.

Response: Thank you for your meticulous suggestion. We agree with the reviewer's comments. Items (5) and (6) in the original conclusion section, which addressed the limitations of this study and future research directions, have been deleted. These contents have been integrated and moved to the last paragraph before the conclusion section (Lines 475-482). Revised as follows:

“It should be noted that this study selected only one representative sample each of subbituminous coal, bituminous coal, and anthracite coal for analysis; therefore, the possible differences among samples of the same coal rank under microwave irradiation cycles were not further examined. In addition, under fixed irradiation conditions of 120 s and 700 W, this work mainly focused on the effect of the number of irradiation cycles on coal pore structure evolution. Since microwave irradiation time and output power are also important factors influencing the modification effect, future studies should further investigate the synergistic effects of irradiation duration, microwave power, and cycle number on the evolution of coal pore structures.”

5. References [15] and [21] are identical, which constitutes a duplicate citation.

Response: Thank you for pointing out this issue. References [15] and [21] are duplicate citations, and we apologize for this oversight. The duplicate references have been removed, and the reference list has been revised (Lines 550-552).

6. The text labels in Fig. 2 should maintain consistent capitalization. Please check the entire manuscript for similar issues and revise accordingly.

Response: Thank you for the suggestion. The capitalization of the labels in Fig. 2 has been standardized, and the entire manuscript has been checked for similar inconsistencies. The revised Figures have been re-uploaded.

Once again, thank you very much for your constructive comments and suggestions, which would help us in depth to improve the quality of the manuscript.

Kind regards.

Yuefang Wang

E-mail address: wyftc@imut.edu.cn

2026.6.4

Attachment Submitted filename: Response_to_Reviewers_auresp_2.docx

# Editor report: Decision Letter 2

**DOI:** 10.1371/journal.pone.0343007.r005

**Authors:** Xinyuan Gao

18 Jun 2026

Effect of microwave irradiation cycles on the pore structures and fractal dimension of coals with different ranks

PONE-D-26-04593R2

Dear Dr. Wang,

We’re pleased to inform you that your manuscript has been judged scientifically suitable for publication and will be formally accepted for publication once it meets all outstanding technical requirements.

Within one week, you’ll receive an e-mail detailing the required amendments. When these have been addressed, you’ll receive a formal acceptance letter and your manuscript will be scheduled for publication.

An invoice will be generated when your article is formally accepted. Please note, if your institution has a publishing partnership with PLOS and your article meets the relevant criteria, all or part of your publication costs will be covered. Please make sure your user information is up-to-date by logging into Editorial Manager at Editorial Manager® and clicking the ‘Update My Information' link at the top of the page. For questions related to billing, please contact billing support.

If your institution or institutions have a press office, please notify them about your upcoming paper to help maximize its impact. If they’ll be preparing press materials, please inform our press team as soon as possible -- no later than 48 hours after receiving the formal acceptance. Your manuscript will remain under strict press embargo until 2 pm Eastern Time on the date of publication. For more information, please contact onepress@plos.org.

Kind regards,

Xinyuan Gao

Academic Editor

PLOS One

Additional Editor Comments (optional):

Reviewers' comments:

# Editor report: Acceptance letter

**DOI:** 10.1371/journal.pone.0343007.r006

**Authors:** Xinyuan Gao

PONE-D-26-04593R2

PLOS One

Dear Dr. Wang,

I'm pleased to inform you that your manuscript has been deemed suitable for publication in PLOS One. Congratulations! Your manuscript is now being handed over to our production team.

At this stage, our production department will prepare your paper for publication. This includes ensuring the following:

* All references, tables, and figures are properly cited

* All relevant supporting information is included in the manuscript submission,

* There are no issues that prevent the paper from being properly typeset

You will receive further instructions from the production team, including instructions on how to review your proof when it is ready. Please keep in mind that we are working through a large volume of accepted articles, so please give us a few days to review your paper and let you know the next and final steps.

Lastly, if your institution or institutions have a press office, please let them know about your upcoming paper now to help maximize its impact. If they'll be preparing press materials, please inform our press team within the next 48 hours. Your manuscript will remain under strict press embargo until 2 pm Eastern Time on the date of publication. For more information, please contact onepress@plos.org.

You will receive an invoice from PLOS for your publication fee after your manuscript has reached the completed accept phase. If you receive an email requesting payment before acceptance or for any other service, this may be a phishing scheme. Learn how to identify phishing emails and protect your accounts at https://explore.plos.org/phishing.

If we can help with anything else, please email us at customercare@plos.org.

Thank you for submitting your work to PLOS ONE and supporting open access.

Kind regards,

PLOS ONE Editorial Office Staff

on behalf of

Dr. Xinyuan Gao

Academic Editor

PLOS One
